# Sodium oligomannate therapeutically remodels gut microbiota and suppresses gut bacterial amino acids-shaped neuroinflammation to inhibit Alzheimer’s disease progression

**DOI:** 10.1038/s41422-019-0216-x

**Published:** 2019-09-06

**Authors:** Xinyi Wang, Guangqiang Sun, Teng Feng, Jing Zhang, Xun Huang, Tao Wang, Zuoquan Xie, Xingkun Chu, Jun Yang, Huan Wang, Shuaishuai Chang, Yanxue Gong, Lingfei Ruan, Guanqun Zhang, Siyuan Yan, Wen Lian, Chen Du, Dabing Yang, Qingli Zhang, Feifei Lin, Jia Liu, Haiyan Zhang, Changrong Ge, Shifu Xiao, Jian Ding, Meiyu Geng

**Affiliations:** 1 Shanghai Green Valley Pharmaceutical Co., Ltd, Shanghai, 201203 China; 2 State Key Laboratory of Drug Research, Shanghai Institute of Materia Medica, Chinese Academy of Sciences, Shanghai, 201203 China; 3 Department of Geriatric Psychiatry, Shanghai Mental Health Center, Shanghai Jiao Tong University School of Medicine; Alzheimer’s Disease and Related Disorders Center, Shanghai Jiao Tong University, Shanghai, 200025 China; 4 Institutional Technology Service Center, Shanghai Institute of Materia Medica, Chinese Academy of Sciences, Shanghai, 201203 China

**Keywords:** Metabolomics, Mechanisms of disease

## Abstract

Recently, increasing evidence has suggested the association between gut dysbiosis and Alzheimer’s disease (AD) progression, yet the role of gut microbiota in AD pathogenesis remains obscure. Herein, we provide a potential mechanistic link between gut microbiota dysbiosis and neuroinflammation in AD progression. Using AD mouse models, we discovered that, during AD progression, the alteration of gut microbiota composition leads to the peripheral accumulation of phenylalanine and isoleucine, which stimulates the differentiation and proliferation of pro-inflammatory T helper 1 (Th1) cells. The brain-infiltrated peripheral Th1 immune cells are associated with the M1 microglia activation, contributing to AD-associated neuroinflammation. Importantly, the elevation of phenylalanine and isoleucine concentrations and the increase of Th1 cell frequency in the blood were also observed in two small independent cohorts of patients with mild cognitive impairment (MCI) due to AD. Furthermore, GV-971, a sodium oligomannate that has demonstrated solid and consistent cognition improvement in a phase 3 clinical trial in China, suppresses gut dysbiosis and the associated phenylalanine/isoleucine accumulation, harnesses neuroinflammation and reverses the cognition impairment. Together, our findings highlight the role of gut dysbiosis-promoted neuroinflammation in AD progression and suggest a novel strategy for AD therapy by remodelling the gut microbiota.

## Introduction

Despite the tremendous efforts made in the treatment of Alzheimer’s disease (AD), the past decades have witnessed the continuous failure of β-amyloid (Aβ)- or tau-centric therapeutic strategies in late-stage clinical trials, which impels the reconsideration of new therapeutic strategy for this complicated disease.^1,2^ Recently, a plethora of studies have shown the dynamic interaction between the intestinal microbiota and host innate and adaptive immune system.^3,4^ The dysbiosis of gut microbiota could jeopardize host immune responses and promote the development of various inflammatory disorders.^5^ This also appears to be the case for AD-associated inflammation. Emerging evidence from both animal and human studies supports the association between dysbiosis of the gut microbiota and the microglia activation during AD development.^6–8^ For example, Minter et al. discovered that perturbations in gut microbial diversity influenced neuro-inflammation and amyloidosis. Antibiotics-treated mice showed a significant decrease in plaque-localized microglial activation positive for IBA-1.^9^ Besides, gut microbiota-produced lipopolysaccharide (LPS) was found in the post-mortem brain samples of AD patients,^10^ and peripheral injection of LPS could promote microglial activation.^11,12^ All the evidence suggests that gut microbiota is likely involved in regulating microglia activation and neuroinflammation in AD.

In addition to the microglia activation, the role of infiltrating peripheral immune cells, such as CD4^+^ and CD8^+^ T cells, etc., in AD-associated neuroinflammation is increasingly appreciated. For example, peripheral type 1 and type 17 T-helper (Th1, Th17) cells have been reported to be associated with releasing of inflammatory cytokines in multiple AD mouse models.^13,14^ Consistent with these evidences, peripheral infiltrated lymphocytes were observed in the brain of both transgenic mouse models and AD patients.^13^ Additionally, in the post-mortem brains of AD patients, both CD4^+^ and CD8^+ ^T cells were detected.^15^

Current understanding of the mechanistic link between the gut and brain in AD progression is still very limited.^16,17^ It remains unknown which types of infiltrated immune cells are functionally involved in AD development.^18,19^ The underlying driving force that promotes peripheral immune cells to infiltrate the brain, leading to the enhancement of the residential neuroinflammation, remains obscure. Moreover, as the importance of microbiota metabolites is starting to unveil, it will be imperative to comprehend the specific metabolites that are involved in linking gut microbiota and brain neuroinflammation in AD progression.

This study aims to investigate the mechanistic linkage between gut microbiota and AD progression, and to explore the potential intervention strategies. To this end, we investigated the association between dynamic changes of gut microbiota and neuroinflammation at different stages of AD development, and probed the possible mechanistic links between intestinal microbiota and neuroinflammation in the aspects of metabolites. In particular, we took the advantage of an oligosaccharide anti-AD drug, designated as GV-971, as a probe to assess the potential intervention approach by targeting the gut microbiota. GV-971 is a mixture of acidic linear oligosaccharides ranging from dimers to decamers (molecular weight up to ~1 kDa). Recently, GV-971 has completed a Phase 3 clinical trial for AD in China and successfully met its primary endpoint in improving cognition impairment (unpublished data).

We have revealed that gut microbiota patterns and amino acid-derived metabolites are important for the infiltration of specific types of immune cells, which drives neuroinflammation during AD progression. GV-971 therapeutically harnesses the abnormal production of amino acids, infiltration of immune cells to the brain, and in turn neuroinflammation via remodelling the gut microbiota. The elucidation of the mechanisms underlying the neuroimmune regulation by microbiota may hold promise for developing novel therapeutics for AD patients.

## Results

### AD progression is associated with the alteration of gut microbiota and immune cell infiltration

**Fig. 1:**
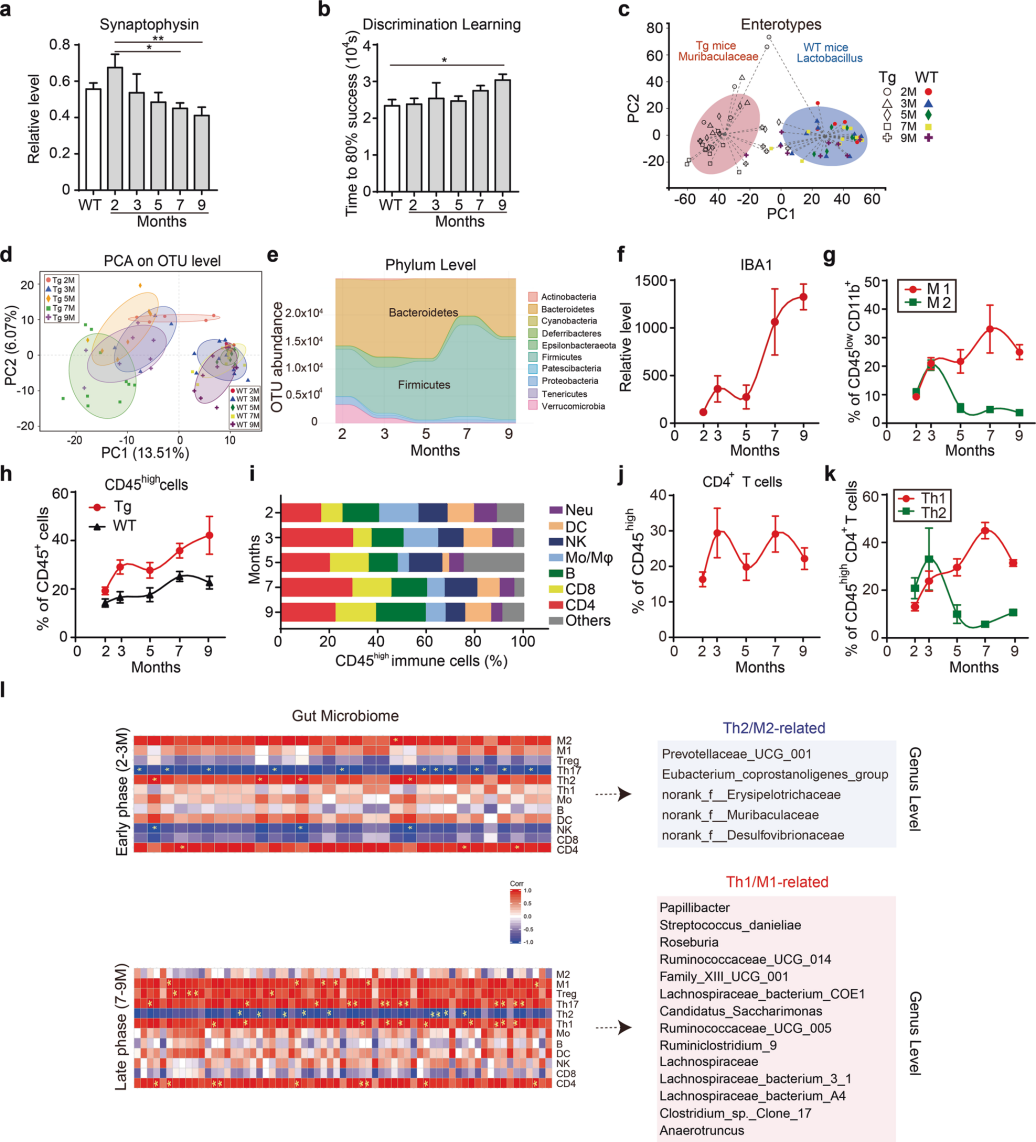
Gut dysbiosis and immune cell changes during disease progression in 5XFAD transgenic (Tg) mice. **a** Changes in the relative RNA expression levels of synaptophysin in the hippocampus of 5XFAD transgenic (Tg) mice at 2, 3, 5, 7 and 9 months and in wild-type (WT) mice at 2 months (*n* = 5–12). The data are presented as mean ± standard error of the mean (mean ± SEM) relative to the expression level of actin. *P *<* 0.05, **P *<* 0.01 by one-way ANOVA (F (5, 43) = 2.952). **b** Changes in the time out of 10^4^ s taken to achieve 80% success (see “Materials and methods”) in a test to evaluate the discrimination learning abilities of 5XFAD transgenic (Tg) mice at 2, 3, 5, 7, and 9 months and wild-type (WT) mice at 2 months (*n* = 4–8). Data are presented as mean ± standard error of the mean (mean ± SEM). *P *<* 0.05 by Student’s t-test. s seconds. **c** Enterotype analysis at the genus level of the gut microbiomes of 5XFAD transgenic (Tg) mice and wild-type (WT) mice at 2-, 3-, 5-, 7- and 9-month old (*n* = 4–10). The separation of gut microbial taxa into a norank genus under the family of *Muribaculaceae* of Tg mice and the genus *Lactobacillus* enterotypes of WT mice is achieved by calculating Bray-Curtis distance based on the relative abundance at the genus level and clustered using PAM (Partitioning Around Medoids). The data are most naturally separated into two clusters, as determined by the Calinski-Harabasz (CH) index and represented using principal coordinate analysis (PCoA). The shapes and colours of the points indicate samples from each individual from various months. The coloured ellipses indicate the 0.95 confidence interval (CI) ranges within each enterotype group. M months. **d** Principal component analysis (PCA) of the gut microbiome composition of WT and 5XFAD transgenic (Tg) mice on the operational taxonomic unit (OTU) level at different time points (*n* = 4–10). The shapes and colours of the points indicate samples from each individual from various months. The coloured ellipses indicate 0.95 confidence interval (CI) ranges within each tested group. M months. **e** Abundance changes of operational taxonomic units (OTUs) in the overall population in the gut microbiome of 5XFAD transgenic (Tg) mice at various months, coloured at the phylum level on a stream graph (*n* = 4–10). The two most abundant phyla, *Bacteroidetes* and *Firmicutes*, are labelled on the graph. Colours indicate different phyla of the gut microbiota. **f** Changes in the positive densities of IBA1 immune-fluorescent staining, reflecting activation of microglial cells in the hippocampus of 5XFAD transgenic (Tg) mice at 2, 3, 5, 7, and 9 months relative to the values of 2-month-old wild-type (WT) mice (*n* = 2–7). The data are presented as mean ± standard error of the mean (mean ± SEM); lines are fitted with a cubic spline. **g** Changes in activated M1 and M2 type microglia detected in the whole-brain homogenates of 5XFAD transgenic (Tg) mice at 2, 3, 5, 7 and 9 months (*n* = 4–8). M1-type microglia (CD45^low^CD11b^+^CX3CR1^+^Siglec-H^+^CD86^+^) and M2-type (CD45^low^CD11b^+^CX3CR1^+^Siglec-H^+^ CD206^+^) microglia were detected by flow cytometry, and their cell counts are presented relative to the frequency of CD45^low^ CD11b^+^ cells. Red points and lines: M1 microglia. Green points and lines: M2 microglia. The data are presented as mean ± standard error of the mean (mean ± SEM); lines are fitted with a cubic spline algorithm. **h** Changes in infiltrating cells (CD45^high^) detected in the whole-brain homogenates of 5XFAD transgenic (Tg) mice (red points and lines) and WT mice (black points and lines) at different time points as detected by flow cytometry (*n* = 4–8). Cell counts are presented relative to the frequency of CD45^+^ cells and formatted as mean ± standard error of the mean (mean ± SEM). Lines are fitted with a cubic spline algorithm. **i** Changes in CD45^high^ cells in 5XFAD transgenic (Tg) mice at different time points (*n* = 4–8). On the barplot, cell counts are presented relative to the frequency of CD45^high^ cells. Colours indicate different subtypes of CD45^high^ cells: Neu, neutrophils; DC, dendritic cells; NK, natural killer cells; Mo/Mϕ ː monocytes and macrophages; B, B cells; Others, unclassified cells. **j** Changes in infiltrating CD4 T cells (CD45^high^CD4^+^) detected in the whole-brain homogenates of 5XFAD transgenic (Tg) mice (red points and lines) at 2, 3, 5, 7 and 9 months as detected by flow cytometry (*n* = 4–8). Cell counts are presented relative to the frequency of CD45^high^ cells and formatted as mean ± standard error of the mean (mean ± SEM). Lines are fitted with a cubic spline. **k** Changes in infiltrating peripheral Th1 and Th2 cells detected in the whole-brain homogenates of 5XFAD transgenic (Tg) mice at 2, 3, 5, 7 and 9 months (*n* = 4–8). Th1 cells (CD45^high^CD4^+^CXCR3^+^) and Th2 cells (CD45^high^CD4^+^CCR4^+^) were detected by flow cytometry, and presented relative to the frequency of CD45^high^CD4^+^ T cells. Red points and lines: Th1 cells. Green points and lines: Th2 cells. The data are presented as mean ± standard error of the mean (mean ± SEM). Lines are fitted with a cubic spline. **l** Correlation between the trend changes of the frequency of brain lymphocytes and the abundance of gut microbiota represented at genus level during the Th2/M2-related stage and Th1/M1-related stage in the early and late phase (2–3 months and 7–9 months), respectively (left panels, *n* = 4–8). Bacteria that are significantly correlated with Th2/M2 or Th1/M1 in 5XFAD mice are listed in the right-hand panels. Squares in red (positive correlation) or blue (negative correlation) with a yellow asterisk (*) indicate significant correlations with *P*-values < 0.05 measured by the Pearson parametric correlation test (see “Materials and methods”). f family, M months, T regulatory cells Treg, Mo monocytes, B B cells, DC dendritic cells, NK natural killer cells

To assess the role of gut microbiota alteration in AD pathogenesis, we used the 5XFAD transgenic (Tg) mouse model, which is widely used in AD study for faithfully recapitulating AD-associated pathological features including the severely accelerated cognitive impairment, amyloid deposition in the 2^nd^ postnatal month, synaptic degeneration in the 4^th^ postnatal month, and behavioural changes in the 6^th^ month.^20–23^ Consistent with these reports,^24–27^ we observed rapid accumulation of Aβ plaque deposition in the cortex and hippocampus beginning from the 3^rd^ postnatal month in Tg mice compared to the age-paired wild-type (WT) mice (Supplementary information, Fig. S1a). Tau phosphorylation in the brain of Tg mice was first found in the 5^th^ month and increased gradually compared to the age-paired WT mice (Supplementary information, Fig. S1b). The synaptophysin expression level in the hippocampus significantly decreased from the 7^th^ to the 9^th^ months, indicative of synaptic degeneration (Fig. 1a), while the behavioural test in Tg mice showed a significant decline in discrimination learning at 9 months of age (Fig. 1b).

We then compared the enterotypes of Tg mice and WT mice at different stages in AD progression using 16S ribosomal RNA (rRNA) gene amplicon sequencing. The sequences were grouped into provisional clusters as operational taxonomic units (OTUs). Enterotype analysis revealed a notable clustering effect in the gut microbiome, with Tg mice clustered into a norank genus under the family of *Muribaculaceae* and WT mice clustered into the *Lactobacillus* genus (Fig. 1c). Using principal component analysis (PCA), which simplifies the complexity in high-dimensional data while retaining trends and patterns upon a series of time points (also see Materials and methods^28^), we revealed a remarkable shift in the gut microbiota composition during AD progression in Tg mice. Hardly any changes were observed in WT mice (Fig. 1d; Supplementary information, Fig. S1c). Using OTUs to track the dynamics of the abundance of different bacterial phyla in Tg mice, we found two distinct changes of gut microbiota profiles in Tg mice during disease progression. At 2 months of age, *Bacteroides, Firmicutes* and *Verrucomicrobia* were the three most abundant bacteria at the phylum level (47.3%, 33.0% and 12.2%, respectively). At 7 months of age, *Firmicutes* became the predominant phylum (62.8%), while the abundance of *Bacteroidetes* and *Verrucomicrobia* markedly decreased, indicating an alteration of the types of bacteria (Fig. 1e). Representative bacteria from Tg mice at each time point were further explored (Supplementary information, Fig. S1d, e and Table S1). These results indicated that the gut microbiota of Tg mice was highly dynamic, in great contrast to that of the WT mice. Similar results were also obtained in the APP/PS1 double transgenic mouse model, another widely used model for AD study (Supplementary information, Fig. S1f, g).^20,29–31^

Previous studies have suggested that gut microbiota is involved in triggering neuroinflammation in the brain.^18,32^ We hypothesized that the observed gut microbiota alteration during AD progression might be associated with neuroinflammation. To test this hypothesis, we evaluated the inflammatory status of Tg mice. Immunostaining of IBA1, a hallmark of microglial activation, in AD mouse brain sections revealed two evident stages of microglial activation, at the 3^rd^ month and the 7–9^th^ months, respectively (Fig. 1f; Supplementary information, Fig. S1h). Given that microglial activation can be categorized into two opposite types, the pro-inflammatory M1 and the neuroprotective M2 subtype,^33^ we also carefully characterized M1 and M2 phenotypes. At early stage of 2-3-month old, both M1 and M2 microglia increased, while in the following months, M1 subtype continued to increase and peaked at 7-9 months, whereas M2 type microglia declined from 3 to 5 month and maintained a low level thereafter (Fig. 1g).

In addition to microglia activation, AD-associated neuroinflammation is known to involve the infiltration of peripheral immune cells.^34–36^ We therefore also analyzed the infiltrating peripheral immune cells in the brain during AD progression. It was observed that the frequency of CD45^high^ cells in the brain was significantly higher in Tg mice than that in WT mice, similar to the result of IBA1 staining (Fig. 1f, h). We further profiled changes of CD45^high^ cell subtypes at a series of time points during AD progression (Fig. 1i). Using the k-means clustering (see Materials and methods), we revealed that the alteration of CD4^+^ T cells, as the major proportion of CD45^high^ cells, closely recapitulated the change of M1 cells (Fig. 1i, j). Infiltrating Th1 and Th2 cells, two major subtypes of CD4^+^ cells, exhibited similar dynamics to that of M1 and M2 microglial cells over the period that we examined (Fig. 1k). It appeared to us that, as the pattern of the gut microbiota shifted, the immune cell population tended to change to a Th1- and M1-dominated state. Similar results were also found in the APP/PS1 model (Supplementary information, Fig. S1i, j) and other AD mouse models by querying public databases (Supplementary information, Fig. S2).^37,38^

We also analyzed the correlation between gut microbiota abundance and brain immune cell frequency at both early and late stages of AD progression. We noted that the abundance changes of bacteria were aligned with the alterations of Th2 and M2 cells at the early stage (2–3 months) (Fig. 1l, top), but correlated with those of Th1 and M1 cells at the late stage (7–9 months) (Fig. 1l, bottom). The bacteria that were significantly interrelated with immune cells were listed in the right panel of Fig. 1l (list of the full names is in Supplementary information, Table S2). Overall, these results indicated that gut bacteria were associated with the infiltration of peripheral immune cells and neuroinflammation occurrence during AD progression.

### Gut microbiota is required for immune cell infiltration and microglial activation in the brain

**Fig. 2:**
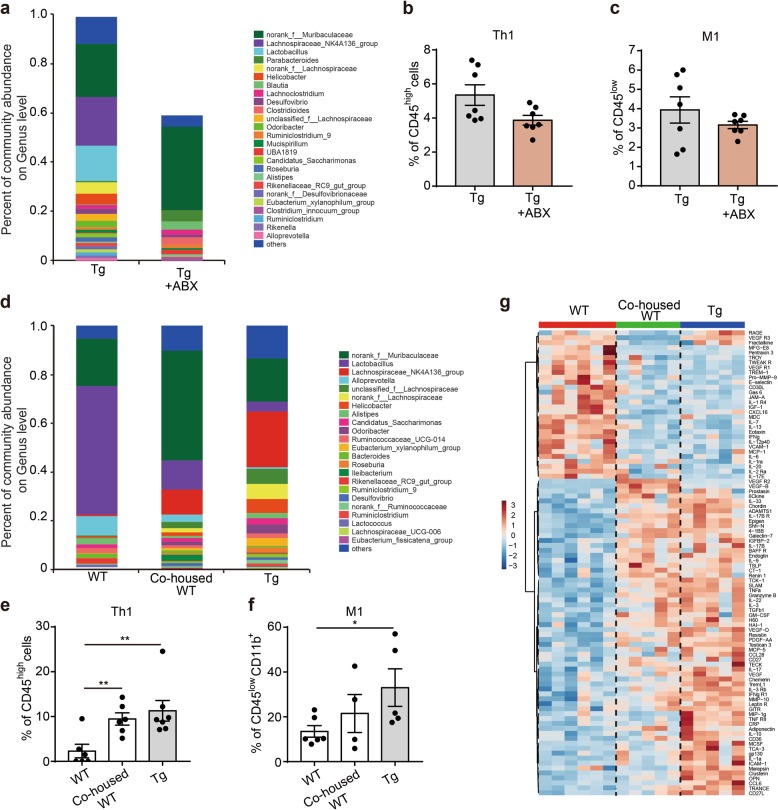
The gut microbiota is required for immune cell infiltration and microglial activation. **a** The effects of five-month oral gavage of antibiotics on the relative abundance of gut microbes in 7-month-old 5XFAD transgenic (Tg) mice (*n* = 5–7). ABX, a cocktail of mixed antibiotics composed of ampicillin (0.1 mg/mL), streptomycin (0.5 mg/mL) and colistin (0.1 mg/mL). Different genera of gut microbes are coloured differently, and their changes in relative abundance are presented on the barplot. The abundance of *Bacteroides* in Tg mice is below 0.01 and was thus not shown in both groups. **b-c** The effects of five-month oral gavage of antibiotics on the frequency of Th1 cells (**b**) and M1-type microglia (**c**) in the brain homogenate of 7-month-old 5XFAD transgenic (Tg) mice. Cell counts of Th1 cells (CD45^high^CD4^+^CXCR3^+^) are presented relative to the frequency of CD45^high^ cells (**b**), while those of M1-type microglia (CD45^low^CD11b^+^CX3CR1^+^Siglec-H^+^CD86^+^) are presented relative to the frequency of CD45^low^ cells (**c**). Both are detected by flow cytometry, *n* = 7, the data are presented as mean ± standard error of the mean (mean ± SEM). **d** The relative abundance of gut microbes at the genus level in WT, co-housed WT and 5XFAD transgenic (Tg) mice (*n* = 6–7). All three groups of mice were at 7-month old. Different colours represent different genera. Co-housed WT: WT mice that were housed with Tg mice. **e-f** Changes in the frequency of Th1 cells (**e**) and M1 type microglia (**f**) in the brain homogenates of 7-month-old WT, co-housed WT and 5XFAD transgenic (Tg) mice (*n* = 4–7). Th1 cells (CD45^high^CD4^+^CXCR3^+^) are presented relative to the frequency of CD45^high^ cells (**e**), while the frequency of M1-type microglia (CD45^low^CD11b^+^CX3CR1^+^Siglec-H^+^CD86^+^) are presented relative to the frequency of CD45^low^CD11b^+^ cells (**f**). Both are detected by flow cytometry. The data are presented as mean ± standard error of the mean (mean ± SEM). *P < 0.05, **P *<* 0.01 by Student’s t-test. **g** Levels of cytokine proteins in the brain homogenates of WT, co-housed WT and 5XFAD transgenic (Tg) mice at 7-month old as detected by a cytokine antibody array (*n* = 5–6). Colours in the heatmap indicate relative cytokine levels; red indicates cytokines that are upregulated, and blue indicates cytokines that are downregulated

To determine whether the gut microbiota change is required for driving peripheral immune cell infiltration and in turn neuroinflammation in AD progression, we used an antibiotic cocktail containing ampicillin (0.1 mg/mL), streptomycin (0.5 mg/mL), and colistin (0.1 mg/mL) to ablate gut microbiota. Antibiotic treatment in Tg mice resulted in a marked reduction in microbial abundance in the gut (Fig. 2a; Supplementary information, Table S3). Along with this change, we observed a reduction in both infiltrating pro-inflammatory Th1 cells (Fig. 2b) and M1 cells (Fig. 2c) in the brain.

Further, these findings were confirmed by a co-housing experiment, in which WT mice were co-housed with Tg mice of the same age for 7 months since birth. The co-housed WT mice displayed the decline in discrimination learning, comparable to that of Tg mice, indicating that the long-term exposure to the Tg bacteria could cause cognitive impairment (Supplementary information, Fig. S3a). Analysis of the gut microbiota change in these mice indicated that the composition of the gut microbiota was quite similar between co-housed WT mice and Tg mice, but significantly different from that of WT mice of the same age under separate housing (Fig. 2d). Moreover, infiltrating Th1 cells between co-housed WT and Tg mice were comparable as well, which were both significantly higher than that of separately housed WT mice (Fig. 2e). Meanwhile, M1 cells were only slightly increased in the co-housed WT mice (Fig. 2f), suggesting that microbiota alteration mainly influenced the infiltrating immune cells rather than the resident immune cells. In line with the immune cell change, cytokine expression in the brain showed a marked similarity between co-housed WT and Tg mice, but distinct from those of WT mice (Fig. 2g; Supplementary information, Table S4).

We also performed faecal microbiota transplantation (FMT) experiments using C57BL/6 WT mice. For C57 WT mice with hippocampal Aβ injection, oral administration of faecal microbiota from 7-month Tg mice resulted in a marked increase in Th1 cells and a reduction in Th2 cells in the brain (Supplementary information, Fig. S3b). Reversely, transplantation of WT feces decreased brain Th1 cells in the recipient Tg mice (Supplementary information, Fig. S3c). Together, these findings suggest that the gut microbiota alteration drives peripheral immune cell infiltration and neuroinflammatory activation in AD progression.

### GV-971 exhibits ameliorative effects on cognitive impairment

**Fig. 3:**
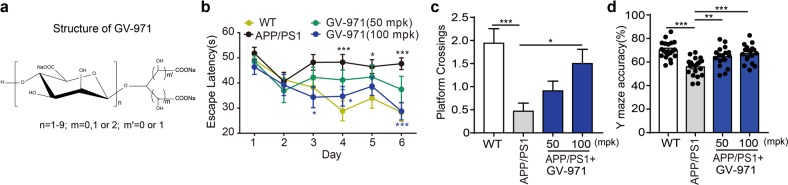
The effects of GV-971 on behaviour changes in APP/PS1 mice models. **a** Structure of GV-971. GV-971 is a mixture of acidic linear oligosaccharides with degrees of polymerization ranging from dimers to decamers with an average molecular weight of approximately 1 kDa. **b** The escape latency time results of the Morris Water Maze (MWM) test as a measurement of spatial learning and memory in APP/PS1 mice. Nine-month-old APP/PS1 mice were treated with 50 mpk and 100 mpk of GV-971 for 3 months until 13-month old. Then, the MWM test for spatial learning and memory abilities were conducted for 6 additional days. During the test, GV-971 was continuously administrated. The escape latency time starting (seconds) was measured as one of the final readouts of the test (see Materials and methods). Higher escape latency time shows that these mice will spend more time to reach the target, which indicates a more severely impaired spatial learning and memory ability (*n* *=* 11–14). The data are presented as mean ± standard error of the mean (mean ± SEM). Black asterisk indicates the comparison between WT and APP/PS1 group. Blue asterisk indicates the comparison between GV-971(100 mpk) treatment and APP/PS1 group. *P < 0.05, ***P *<* 0.001 by two-way ANOVA. **c** The number of platform-site crossovers in MWM test as a measurement of spatial learning and memory in APP/PS1 mice. Nine-month-old APP/PS1 mice were treated with 50 mpk and 100 mpk of GV-971 for 3 months until 13-month old. Then, the MWM test for spatial learning and memory abilities were conducted for 6 additional days. During the test, GV-971 was continuously administrated. The number of platform-site crossovers was measured as the other readout of the test (see Materials and methods). Larger numbers of platform-site crossovers indicate less severely impaired spatial learning and memory ability (*n* **=** 11–17). *P < 0.05, ***P *<* 0.001 by one-way ANOVA (F (3, 55) = 6.542). **d** The accuracy of spatial working memory as tested using the Y maze in APP/PS1 mice. Nine-month-old APP/PS1 mice were treated with 50 mpk and 100 mpk of GV-971 for 3 months until 12-month old. Then the Y maze test was conducted. During the test, GV-971 was continuously administrated. The accuracy of the Y maze was the ratio between the correct alternation and the total alternation (see “Materials and methods”). Higher accuracy indicates less severely inpaired working memory abilities. (*n* = 17–20). The data are presented as mean ± standard error of the mean (mean ± SEM). **P < 0.01, ***P < 0.001 by one-way ANOVA (F (3, 71) = 12.39)

The essential role of gut microbiota in AD progression revealed herein may suggest the therapeutic implications by the intervention of gut microbiota. To test this hypothesis, we took advantage of GV-971 (Fig. 3a). In 9-month-old APP/PS1 mice treated with GV-971 for three months until 13-month old, GV-971 exhibited significant ameliorative effect on the cognitive impairment, as shown by the enhanced spatial learning and memory performance of APP/PS1 mice in both training trial (Fig. 3b) and probe trial (Fig. 3c) in the Morris Water Maze (MWM) task. GV-971 also significantly improved the mice performance in Y maze (Fig. 3d). Recently, GV-971 has shown the therapeutic effect on reversing cognition impairment in AD patients in a 36-week multi-center, randomized, double-blind, placebo-controlled Phase 3 clinical trial in China. Given that carbohydrates, in the forms of monosaccharide or oligosaccharide, are the primary nutrient source for bacteria and have shown diverse modulatory effects on bacteria,^39–42^ we were interested to explore whether GV-971 could affect the gut microbiota.

### GV-971 alleviates neuroinflammation by shaping the gut microbiota

**Fig. 4:**
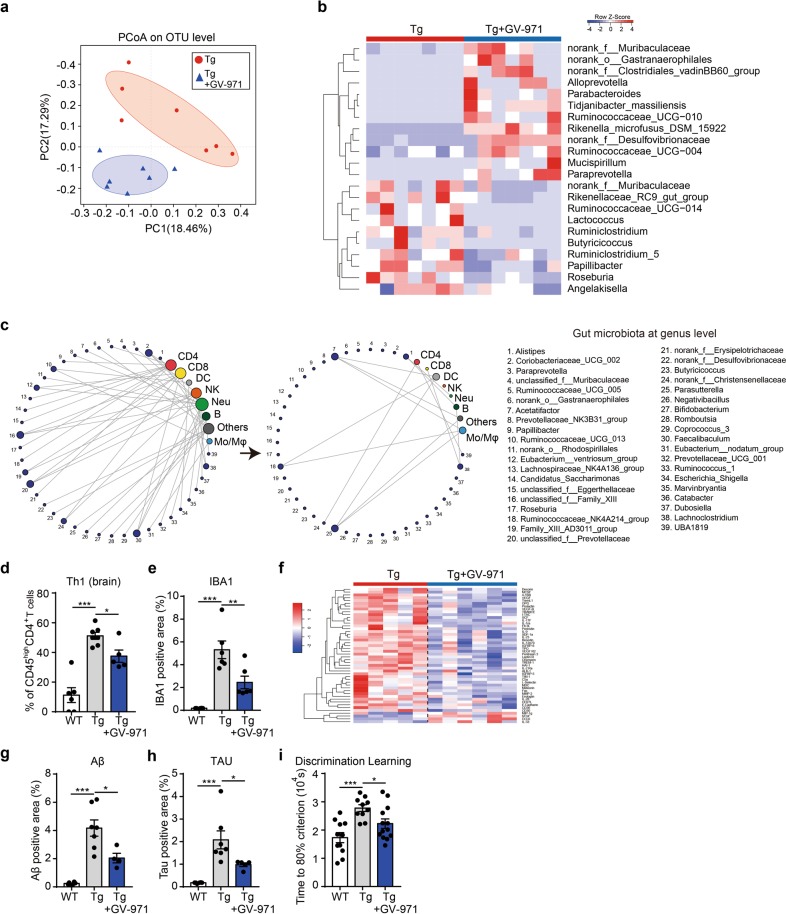
GV-971 alleviates neuroinflammation by reconditioning the gut microbiota. **a** Principal coordinate analysis (PCoA) of the gut microbiome composition on the operational taxonomic unit (OTU) level based on the Bray-Curtis distance for 5XFAD (Tg) mice and GV-971-treated Tg mice at 7-month old (*n* = 7). The shapes and colours of the points indicate samples from each individual. Coloured ellipses indicate 0.95 confidence interval (CI) ranges within each tested group. PC principal component. **b** Heatmap of significant gut microbiota changes represented at the genus level between 5XFAD (Tg) mice and GV-971-treated Tg mice at 7-month old (*n* = 7). Colours on the heatmap indicate the relative abundance of gut microbiota; red indicates bacteria that are upregulated, and blue indicates bacteria that are downregulated. Gut microbiota with significant changes were chosen using a Wilcoxon rank-sum two-tailed test with *P*-value that is less than 0.05 between Tg and GV-971-treated groups. f family, o order. **c** Changes in correlational links between the gut microbiome at the genus level (designated with numbers near the purple circles) and brain lymphocytes (other coloured circles) before (left) and after (right) oral gavage of GV-971 in 7-month-old 5XFAD (Tg) mice (*n* = 5–7). Lines represent either significant (*P*-value < 0.05) positive or negative correlation (Pearson coefficient). The size of each circle of immune cells are positively related to the links connected to this circle. The right side lists the name of each gut microbiome, also see “Materials and methods”. f family, o order, DC dendritic cells, NK natural killer cells, Neu neutrophils, B B cells, Mo/Mϕ monocytes and macrophages. **d** Effect of GV-971 treatment on the frequency of brain Th1 cells in 5XFAD (Tg) mice at 7 months old (*n* = 5–7). Th1 cell counts (CD45^high^CD4^+^CXCR3^+^) are presented relative to CD45^high^CD4^+^ T cell counts detected by flow cytometry. The data are presented as mean ± standard error of the mean (mean ± SEM). *P *<* 0.05, ***P < 0.001, by Student’s t-test. **e** Effect of GV-971 treatment on the positive signal density of IBA1 immunofluorescent staining detected in hippocampal slices from 5XFAD (Tg) mice at 7-month old, reflecting activation of microglial cells (*n* = 4–6). The data are presented as mean ± standard error of the mean (mean ± SEM). **P *<* 0.01, ***P *<* 0.001, by one-way ANOVA (F (2, 15) = 21.94). **f** Effect of GV-971 treatment on levels of cytokine proteins in the brain homogenates of 5XFAD (Tg) mice at 7-month old as detected by a cytokine antibody array (*n* *=* 5–6). Colours on the heatmap indicate relative cytokine levels; red indicates cytokines that are upregulated, and blue indicates cytokines that are downregulated. **g, h** Effect of GV-971 on Aβ-positive area (**g**) and tau-positive area (**h**) in the hippocampus of 5XFAD (Tg) mice at 7-month old, evaluated in brain slices (*n* = 4–7). The data are presented as mean ± standard error of the mean (mean ± SEM). For Aβ analysis: *P *<* 0.05, ***P *<* 0.001 (F (2, 14) = 22.78). For tau analysis: *P *<* 0.05, ***P *<* 0.001 (F (2, 15) = 13.06) by one-way ANOVA. **i** Effects of GV-971 on the time out of 10^4^ sec (s) taken to achieve 80% success (see “Materials and methods”) in a test to evaluate the discrimination learning abilities of 5XFAD (Tg) mice at 7-month old (*n* = 10–13). Time means the time to reach the 80% performance level (seconds); the longer it takes, the severer the cognitive impairment is (see “Materials and methods”). *P *<* 0.05, ***P *<* 0.001 by One-way ANOVA (F(2,31) = 9.751). The concentration of GV-971 was 100 mpk for all of the above results

Intriguingly, one-month oral administration of GV-971 in Tg mice beginning from 7-month-old age markedly altered the composition of gut microbiota in a bi-directional way (Fig. 4a, b; Supplementary information, Fig. S4a and Table S2). In line with the gut microbiota alteration, GV-971 treatment in Tg mice disrupted the correlation previously seen between brain lymphocytes and gut bacterial change (Fig. 4c; Supplementary information, Fig. S4b, c and Table S2), decreased brain Th1 cells (Fig. 4d), significantly reduced microglial activation (Fig. 4e), and decreased multiple brain cytokines levels (Fig. 4f; Supplementary information, Table S4). In parallel, GV-971 treatment significantly reduced the Aβ plaque deposition, tau phosphorylation, and ameliorated the decline in discrimination learning in Tg mice (Fig. 4g–i; Supplementary information, Fig. S4d).

Further, we did FMT, transplanting feces of GV-971-treated Tg mice to the recipient C57BL/6 WT mice which were intraventricularly injected with aggregated Aβ. Feces of GV-971-treated Tg mice resulted in decreased Th1 cells in the brain of recipient mice as compared to that of Tg mice without GV-971 treatment (Supplementary information, Fig. S4e). Consistently, antibiotic treatment impaired the effect of GV-971 on gut microbiota, Th1 cells, IBA1 levels and cytokine expression in the APP/PS1 mouse model (Supplementary information, Fig. S4f-j). In addition, treatment with oligo-guluronic acid (PG), an epimer of GV-971, or with polymannuronate sulfate, polyguluronate sulfate, heparin, heparan sulfate did not show the therapeutic effects (data not shown), indicating that the sugar backbone of GV-971 may account for its specific impact on gut microbiota. All these data suggested that GV-971 could alleviate neuroinflammation and cognition decline via modulating gut dysbiosis.

### GV-971 inhibits neuroinflammation by regulating amino acid metabolism

Emerging evidence has highlighted the role of gut microbiota-associated metabolites in influencing the host immune system.^43^ To test the possible involvement of metabolites in immune modulation, the supernatant of in vitro-cultured feces from 7-month-old Tg mice^44–46^ was added to naïve CD4^+^ T cell culture (see Materials and methods), which stimulated the differentiation of naïve CD4^+^ T cells to Th1 and Th2 cells. In contrast, fecal supernatant of Tg mice treated with GV-971 inhibited Th1 differentiation and promoted Th2 differentiation (Supplementary information, Fig. S5a).

**Fig. 5:**
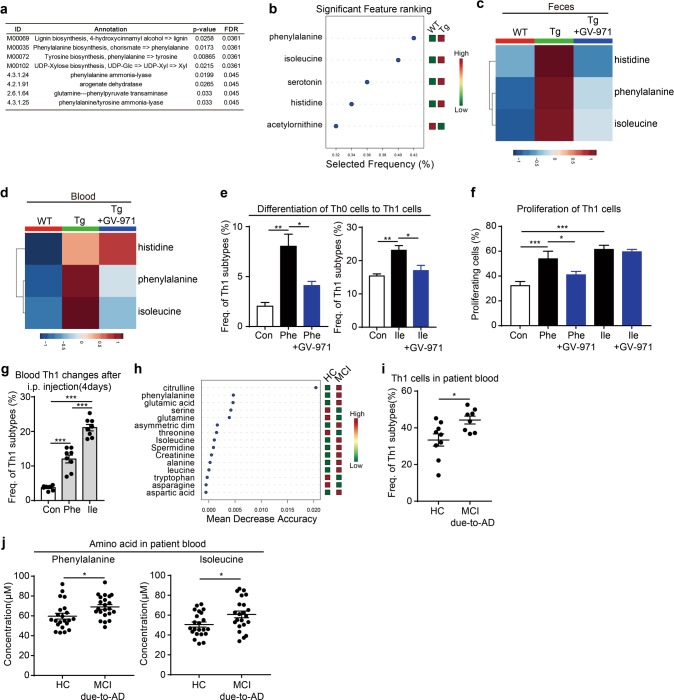
GV-971 inhibits neuroinflammation by harnessing amino acid metabolism. **a** Pathway enrichment analysis of faecal metabolites in 7-month-old 5XFAD (Tg) mice with or without GV-971 treatment (100 mpk) using MBROLE (*n* = 6–8). A partial ist of the enrichment results is presented with KEGG modules and KEGG enzyme interactions which have been screened using a criterion of FDR-adjusted *P*-value < 0.05. **b** Lists of the most important blood amino acids of the random forest model ranked from most to least important between WT (2 m-9 m) and Tg (2 m-9 m) group from a ROC curve analysis. Red indicated high concentration, green indicated low concentration. (*n* = 30 for WT, *n* = 26 for Tg). **c** Changes in histidine, phenylalanine and isoleucine levels in the feces of WT, 5XFAD mice (Tg), and GV-971-treated Tg mice (100 mpk) (*n* = 6–11) at 7-month old. Colours in the heatmap indicate relative metabolite levels; red indicates metabolites that are upregulated, and blue indicates metabolites that are downregulated. **d** Changes in histidine, phenylalanine and isoleucine levels in the blood of WT, 5XFAD mice (Tg), and GV-971-treated Tg mice (100 mpk) (*n* = 6–7) at 7-month old. Colours in the heatmap indicate relative metabolite levels. Red indicates metabolites that are upregulated, and blue indicates metabolites that are downregulated. **e** The effects of GV-971 on the differentiation of naïve CD4^+^ T cells (Th0 cells) to Th1 cells induced by phenylalanine and isoleucine in vitro. Naïve CD4^+^ T cells were cultured for 5 days with/without GV-971 in the presence of phenylalanine (1 mM) or isoleucine (1 mM). The frequency of Th1 (CD4^+^IFN-γ^+^) cells was tested by flow cytometry (see Materials and methods). GV-971 was used at a final concentration of 100 µg/mL. The data are presented as mean ± standard error of the mean (mean ± SEM); *n* = 3 replicates per group, one of three replicated results was represented. Left, *P *<* 0.05, **P *<* 0.01 by one-way ANOVA (F (2, 6) = 15.64). Right, *P *<* 0.05, **P *<* 0.01 by one-way ANOVA (F (2, 6) = 10.35). **f** The effects of GV-971 on the proliferation of Th1 cells induced by phenylalanine and isoleucine. Th1 cells were stained with CellTrace and cultured for 4 days with/without GV-971 in the presence of phenylalanine (1 mM) and isoleucine (1 mM). The density of CellTrace fluorescence in Th1 (CD4^+^IFN-γ^+^) cells was tested by flow cytometry (see Materials and methods). GV-971 was used at a final concentration of 100 µg/mL. The data are presented as mean ± standard error of the mean (mean ± SEM), *n* = 3 replicates per group, one of three replicated results was represented. *P *<* 0.05, ***P *<* 0.001 by one-way ANOVA (F (4, 9) = 28.34). Phe, phenylalanine; Ile, isoleucine. **g** Frequency of blood Th1 cell changes in C57 mice after 4-day intraperitoneal (i.p.) injection of phenylalanine and isoleucine (*n* = 8). ***P *<* 0.001 by one-way ANOVA (F (2, 21) = 101.8). **h** Random forest classification of amino acid changes in healthy controls (HC) and mild cognitive impairment (MCI) due to AD patients. The amino acids are ranked by mean decrease in classification accuracy (first cohort, *n* = 9 for MCI due to AD, *n* = 18 for HC). Red indicated high concentration, green indicated low concentration. **i** Frequency of Th1 cells in the blood of healthy controls (HC) and mild cognitive impairment (MCI) due to AD patients (first cohort, *n* = 8 for MCI due to AD, *n* = 9 for HC). *P *<* 0.05 by Student’s t-test. **j** Levels of phenylalanine and isoleucine in the blood of healthy controls (HC) and mild cognitive impairment (MCI) due to AD patients (second cohort, *n* = 22 for both groups). *P < 0.05 by Student’s t-test

We next employed a non-targeted metabolomics technique to characterize the fecal metabolome. A total of 11289 metabolites were identified in faecal samples from WT, Tg and GV-971-treated Tg mice (Supplementary information, Fig. S5b-c). Among those metabolites matched to database (Supplementary information, Fig. S5d-e), the abundance of 124 metabolites, as annotated by METLIN database, was significantly changed in Tg mice as compared to that in WT mice (Supplementary information, Fig. S5f and Table S5). These altered metabolites were further annotated with the Human Metabolome Database (HMDB) and the Kyoto Encyclopaedia of Genes and Genomes (KEGG), yielding a total of 31 metabolites that were differentially regulated among WT, Tg and GV-971-treated Tg mice, which could be matched to all three databases (Supplementary information, Fig. S5g). Pathway enrichment analysis of these metabolites using MBROLE or MetaboAnalyst^47,48^ further revealed significant changes in amino acid-related metabolic pathways and enzymes, especially phenylalanine-related pathways (Fig. 5a).

We therefore chose to focus on amino acids for further study. Plasma concentrations of a total of 36 amino acids were screened in both WT and Tg mice (Supplementary information, Table S6). Classification of these amino acids using a random forest algorithm^49^ ranked phenylalanine as the top hit that was different between WT and Tg, followed by isoleucine, serotonin, histidine and acetylornithine (Fig. 5b; Supplementary information, Fig. S5h), suggested their strong correlation with the disease progression. We then examined the concentration of the selected amino acids in the faecal and blood samples in GV-971-treated or untreated Tg mice, and compared it with that of WT mice. We found that the concentrations of phenylalanine and isoleucine were significantly higher in the feces of Tg mice than those of WT mice, and GV-971 treatment significantly reduced their concentrations to a level comparable to that of WT mice (Fig. 5c). A similar change in the concentration of phenylalanine and isoleucine was detected in blood (Fig. 5d). To test whether the elevation of these amino acids resulted from gut microbiota change, we examined the concentration of amino acids in FMT study. Feces from 2-month-old WT mice could significantly reduce the concentration of phenylalanine and isoleucine in Tg mice (Supplementary information, Fig. S5i). Likewise, in co-housed WT mice across various months of age, phenylalanine and isoleucine concentrations in blood were also elevated, comparable to that of Tg mice (Supplementary information, Fig. S5j).

Amino acids could be taken by immune cells through specific transporters, driving immune cell differentiation and proliferation.^50^ We examined the expression levels of *Slc7a5*, the transporter of phenylalanine and isoleucine, in Th1 cells and found that *Slc7a5* was expressed in Th1 cells (data not shown). Furthermore, incubation of Th1 cells with ^13^C-labelled phenylalanine revealed the uptake of phenylalanine by Th1 cells, which could be blocked by a pharmacological inhibitor of SLC7A5 (Supplementary information, Fig. S5k), suggesting that Th1 cells are prone to phenylalanine and isoleucine uptake.

To assess the direct effects of phenylalanine and isoleucine on T cell differentiation, naïve CD4^+^ T cells were exposed to either phenylalanine or isoleucine with or without GV-971 for 4 days and Th1 cells were assessed by Flow cytometry (FACS). We observed the significantly enhanced Th1 cell differentiation exposed to either phenylalanine or isoleucine, which was inhibited by GV-971 treatment (Fig. 5e). Next, we evaluated the direct influence of phenylalanine and isoleucine on Th1 cell proliferation. We found that the proliferation of Th1 cells was significantly promoted by phenylalanine and isoleucine, and GV-971 treatment inhibited phenylalanine-induced Th1 cell proliferation, but barely changed isoleucine’s effect (Fig. 5f, blue column). Furthermore, we treated WT mice with intraperitoneal injection of phenylalanine and isoleucine and observed that Th1 cell frequency in the blood was significantly increased (Fig. 5g).

Finally, we explored whether the above findings could be recapitulated in MCI due to AD (see “Materials and methods” for definition of MCI used in this study) patients. Indeed, phenylalanine and isoleucine concentrations as well as Th1 cell frequency in the blood of MCI due to AD subjects were significantly higher than those in the age-matched healthy counterparts (Fig. 5h, i). Moreover, the increased levels of both phenylalanine and isoleucine in the blood were also confirmed in another small MCI due to AD cohort (Fig. 5j), indicating that the abnormal accumulation of phenylalanine and isoleucine and elevation of Th1 frequency in the blood might function as a signature to differentiate the MCI due to AD patients from the healthy subjects.

## Discussion

In this study, we revealed that dysbiosis of the gut microbiota is required for the infiltration of various peripheral immune cells, including CD4^+^ and CD8^+^ T cells, B cells, natural killer (NK) cells, neutrophil, dendritic cells (DCs) and monocytes, to the brain. Among them, Th1 cells were particularly noted for the close association with the M1 microglia activation during AD progression. Given the well-recognized functional crosstalk between Th1 and M1 microglia in the brain,^51^ we propose that gut dysbiosis promotes Th1 cell infiltration to allow their local crosstalk with the M1 microglia and in turn triggers the microglia differentiation towards a pro-inflammatory state.

This mechanistic insight is strengthened by a series of findings obtained in this study. Firstly, a dynamic shift of gut microbiota composition during AD progression is significantly correlated with the elevation of Th1 cell infiltration. Secondly, ablation of the gut microbiota by antibiotic treatment blocked Th1 cells infiltration and M1 microglia activation in AD mice. Thirdly, both long-term exposure to faecal bacteria (co-housing experiment) and FMT of faecal bacteria from AD mice markedly enhanced Th1 cell infiltration and M1 microglia activation in WT mice, while reverse FMT of WT mice feces into Tg mice reduced Th1 cells of the recipient Tg mice. Our findings collectively highlighted the gut microbiota as a driving factor in promoting Th1/M1 microglia-predominated neuroinflammation in AD progression. Interestingly, gut-derived inflammatory immune cells such as Treg and γδT were also reported to play roles in ischemic brain injury^52^ and dietary salt-induced cognitive impairment,^53^ which further supports the functional link between gut microbiota and neuroinflammation and brain functions.

In our study, we did not observe any sex-dependent changes in terms of gut microbiota composition or brain M1/Th1 cell abundance, which seemed different from recent findings showing sex-dependent changes of microglia and Aβ deposition under antibiotic treatment.^54^ We speculate that this discrepancy might result from the different experimental settings, including mouse models (APP/PS1-21 vs. 5XFAD), antibiotic treatment periods at different ages of mice and readout parameters.

The mechanistic relationship regarding gut microbiota dysbiosis and neuroinflammation in AD remains unclear, though advancements have recently been made in other neurological disorders including Parkinson’s disease (PD). Previous studies suggest the direct or indirect involvement of bacterial metabolites, in particular, short-chain fatty acid (SCFA) and bile acids^32,55^ in some neurological disorders. In this study, we detected many metabolites significantly altered in AD mice compared to WT mice. Among them, the most significant change occurred in amino acids, particularly those in phenylalanine-related pathways. We confirmed that the abundance of phenylalanine and isoleucine was increased in the feces and blood of AD mice compared to that of WT mice. Functional assessment both in vitro and in vivo revealed the role of phenylalanine and isoleucine in promoting both differentiation and proliferation of peripheral inflammatory Th1 cells. These findings highlight the role of the abnormal production of phenylalanine and isoleucine by gut microbiota in provoking Th1 cell-dominated neuroinflammation. Consistent with this notion, we detected higher concentration of phenylalanine/isoleucine and Th1 cell frequency in the blood of MCI due to AD patients compared with that of the healthy control cohort.

**Fig. 6:**
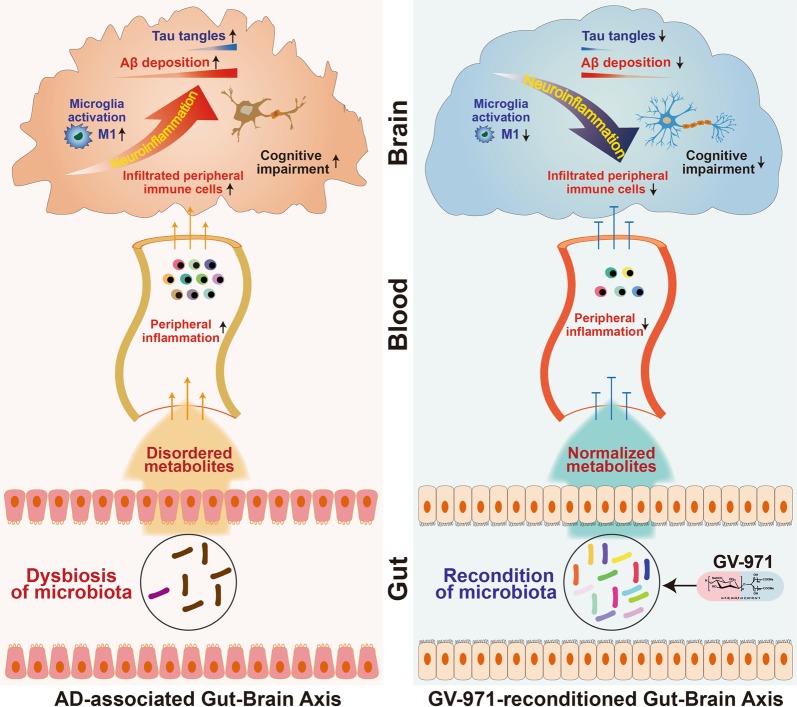
Schematic diagram of gut-brain axis in AD progression and the intervention strategy. Along with Aβ deposition and tau phosphorylation, the alteration of the gut microbiota during AD progression causes metabolic disorder. The abnormal metabolites production provoke peripheral inflammation, increases the brain infiltration of immune cells which crosstalk with M1 microglial cells in the brain, resulting in pathological neuroinflammation and cognitive impairment (left panel). Oral administration of GV-971 reconditions the gut microbiota, normalizes disordered metabolites, reduces the peripheral immune cell infiltration to the brain, resolves neuroinflammation, and reduces Aβ deposition and tau phosphorylation, leading to ultimate improvement of cognitive functions (right panel)

All these findings allowed us to propose a conceptual advancement in understanding the mechanism of AD pathogenesis. AD is not only an Aβ-driven brain disease. Its development also requires the systematic interaction between gut, brain and the intermediate inflammatory factors. In the context of Aβ deposition, the altered gut microbiota composition during AD progression causes an abnormal elevation of amino acids, phenylalanine and isoleucine in particular. These amino acids promote the infiltration of peripheral Th1 cells into the brain via blood circulation. The infiltrating peripheral Th1 cells may locally crosstalk with M1 microglial cells in the brain, resulting in pathological neuroinflammation and cognitive impairment (Fig. 6). These mechanistic insights into AD pathogenesis may provide a novel therapeutic solution by reconstituting the gut microbiota to favour anti-neuroinflammation responses.

Emerging data have shown that poly- or oligosaccharides have advantages to modulate gut microbiota.^56^ The oligomannate sodium GV-971 is a carbohydrate-based anti-AD drug that has been demonstrated to reverse cognitive impairment in mild-to-moderate AD patients in a recently completed phase 3 clinical trial in China. GV-971 is well tolerated with a safety profile comparable to the placebo control. In this study, we found that GV-971 effectively reconditioned gut microbiota, decreased the concentration of phenylalanine and isoleucine in the feces and blood, and reduced Th1-related neuroinflammation in the brain. Of note, GV-971-treated Tg feces could largely mimic the therapeutic effects of GV-971 treatment per se, while antibiotic treatment abrogated the therapeutic effects of GV-971. These findings provided important evidence showing that the therapeutic effect of GV-971 is primarily via the reconstitution of gut microbiome. As such, GV-971 may provide us an appealing approach of microbiota-centric anti-AD strategies worthy of future investigations.

Our findings may have translational implications for AD diagnosis and therapy. The combination of specific bacteria (e.g., Th1/M1-associated bacteria), amino acids (e.g., phenylalanine and isoleucine) and brain infiltrating immune cell composition (e.g., Th1 dominant) could be used as an early diagnostic biomarker for MCI due to AD patients, which merits further validation in large cohorts of AD patients. More importantly, the identified microbiota-centric anti-AD effect of GV-971 will open a new therapeutic avenue for AD treatment through remodelling gut microbiota, and guide the future development of effective therapies by exploring the enormous chemical space of carbohydrates.

Meanwhile, our study has several limitations. Though we have revealed the potential role of gut microbiota in the pathogenesis of AD, future studies to identify the key bacteria strains that may account for metabolites and neuroinflammation changes will be critical. Beyond the two key amino acids that have been fully studied in this study, further exploration will be required to understand other mechanistic links connecting gut microbiota and neuroinflammation. Future studies to address these key issues may help gain a better understanding of the pathogenesis of AD and explore the broader therapeutic potential of GV-971 in clinical treatment.

## Materials and methods

### GV-971

GV-971 is a mixture of oligosaccharides with the degree of polymerization (dp) from 2 to 10. The non-clinical study showed that GV-971 penetrates the blood-brain-barrier (BBB) in its original form. Type 1 glucose transporter (GLUT1) was identified as one of the transporters accounting for its penetration mechanism of BBB. GV-971 could directly bind to multiple subregions of Aβ to inhibit Aβ fibril formation and destabilize the preformed fibrils into non-toxic monomers. Through targeting Aβ, GV-971 promoted microglia-mediated Aβ phagocytosis in vitro, and reversed cognition impairment in multiple AD models. All the preclinical pharmacology, PD/PK and toxicology studies were completed.

GV-971 exhibited a safe and well-tolerated profile in both phase 1 and phase 2 trials. In the recently completed phase 3 trial (NCT02293915), GV-971 was demonstrated to meet the primary endpoint, with statistic significance (p < 0.001). No serious adverse events were observed, with similar incidence rate between GV-971 and placebo group (unpublished data). We are currently applying for NDA filing for GV-971 in China.

### Animals

5XFAD (Tg) mice and co-housed WT mice (the corresponding WT mice generated by mating Tg mice and C57 mice) were bred in the same cage after birth. The WT mice (C57) were bred in separate cages to avoid microbiota cross transfer. All the mice were maintained in a room at 23 °C under a 12-hour (h) light-dark cycle. Mice were randomly allocated to different groups before treatment. For time course analysis of Tg mice, male and female Tg mice were sacrificed at 2-, 3-, 5-, 7- and 9-month old. Mouse brains were collected and stained for immune cell analysis and cytokine analysis. Before the mice were sacrificed, feces were collected for gut microbiota analysis. For GV-971 treatment, at 7-month old, Tg mice were treated with GV-971 at 100 mg/kg dose by oral gavage once daily for one month based on 450 mg twice per day in the Phase 3 trial. A behavioral test was performed to monitor cognitive activity after the last treatment. Then, the mouse brains and feces were used for different analyses. For behavior test, Tg mice and WT mice at different months as well as mice after treatment were tested by discrimination learning, as reported previously.^57^ For phenylalanine and isoleucine intraperitoneal injection treatment, 4-month-old C57 mice were treated with phenylalanine or isoleucine at 50 mg/kg for 4 days.

For Morris water maze (MWM) and Y maze tests, APP/PS1 mice were dosed orally with GV-971 at 50 and 100 mg/kg/day beginning at 9 months. Two tests were carried out at 13 months. For APP/PS1 mice time point analysis, feces were collected at various months of age before we sacrificed the mice for immune cell analysis. The mice and age-matched wild-type mice were sacrificed at 3-, 9-, and 14-month old for immune cell analysis. Mouse brains and blood were collected for different analyses. For APP/PS1 mice treated with GV-971 and antibiotics, 6-month-old mice were treated with GV-971 at 50 mg/kg by oral gavage once daily with or without antibiotic treatment for 2 months. The animal experiments were approved by the ethical committee of Shanghai SIPPR-Bk Lab Animal Co., Ltd (Number: 2016002) and by the Institutional Animal Care and Use Committee at Shanghai Institute of Materia Medica, China.

### Behavioral tests

The MWM test is used to measure spatial learning and memory according to a protocol published previously.^58^ Briefly, the mice underwent 6-day acquisition experiments, and each mouse performed 3 trials each day. The animals were released into the water at the desired start position, and the latency to find the platform was timed. On the 7^th^ day, the platform was removed, and the mice were allowed to swim for 60 s. The trace and the number of platform-site crossovers were recorded using a video camera.

Working memory was assessed by the Y maze according to the literature with some modifications.^58^ The Y maze was composed of three identical arms (A, B, C) with different cues. Mice were placed in the start arm (A) and the sequence of explored arms was recorded (such as ABCBA). The total number of arm entries and alternation behavior were recorded using a video camera. The accuracy of the Y maze was the ratio between the correct alternation and the total alternation.

Discrimination learning. 5XFAD mice and WT mice at different months as well as mice after treatment were tested by Cognition Wall discrimination spatial learning task, as reported previously.^57^ Mice were habituated to individual cages one week before the test. Approximately, 6 h before the test (10:00 am), the mice were housed in the automated PhenoTyper (HomeCage) to habituate to the cage. During the habituation phase, the mice had free access to water but limited access to food. The task began at 4:00 pm and continued during the night for 16 h. During the task, mice need to learn to enter the Cognition Wall through the left entrance (three entrances were offered) to obtain food (one food pellet was rewarded for every fifth entry through the left entrance). The total time needed to reach a criterion of 80% correct, computed as a moving window with window size equal to 30 (i.e., 24 correct entries out of the 30 last entries), was used as a measure during the task. All mouse movements were recorded by a computerized tracking system (Noldus, Ethovision). More time to reach the 80% correct criterion reflects less discriminative ability.

### Faecal sample DNA extraction, PCR amplification and sequencing

All faecal samples were frozen at −80 °C before DNA extraction and analysis. The following steps were conducted by Majorbio Bio-Pharm Technology Co., Ltd. (Shanghai, China). Microbial DNA was extracted from faecal samples using the E.Z.N.A.® Soil DNA Kit (Omega Bio-Tek, Norcross, GA, U.S.) according to the manufacturer’s protocols. The final DNA concentration and purification were determined by a NanoDrop 2000 UV-vis spectrophotometer (Thermo Scientific, Wilmington, USA), and DNA quality was checked by 1% agarose gel electrophoresis. The V3-V4 hypervariable regions of the bacterial 16S rRNA gene were amplified with primers 338 F (5′-ACTCCTACGGGAGGCAGCAG-3′) and 806 R (5′-GGACTACHVGGGTWTCTAAT-3′) by a thermocycler PCR system (GeneAmp 9700, ABI, USA). PCR reactions were conducted using the following program: 3 min (min) of denaturation at 95 °C, 27 cycles of 30 sec (s) at 95 °C, 30 s for annealing at 55 °C, and 45 s for elongation at 72 °C, and a final extension at 72 °C for 10 min. PCR reactions were performed in triplicate in a 20 μL mixture containing 4 μL of 5 × FastPfu Buffer, 2 μL of 2.5 mmol/L dNTPs, 0.8 μL of each primer (5 μmol/L), 0.4 μL of FastPfu Polymerase and 10 ng of template DNA. The resulting PCR products were extracted from a 2% agarose gel and further purified using the AxyPrep DNA Gel Extraction Kit (Axygen Biosciences, Union City, CA, USA) and quantified using QuantiFluor™-ST (Promega, USA) according to the manufacturer’s protocol. Purified amplicons were pooled in equimolar and paired-end sequenced (2 × 300) on an Illumina MiSeq platform (Illumina, San Diego, USA).

### Processing of sequencing data

The following steps were conducted by Majorbio Bio-Pharm Technology Co., Ltd. (Shanghai, China). Raw fastq files were demultiplexed, quality filtered by Trimmomatic and merged by FLASH based on the following criteria: (i) The reads were truncated at any site that received an average quality score < 20 over a 50 bp sliding window. (ii) The primers were exactly matched, allowing a 2-nucleotide mismatch, and reads containing ambiguous bases were removed. (iii) Sequences with overlaps of longer than 10 bp were merged according to their overlap sequence. Operational taxonomic units (OTUs) were clustered with a 97% similarity cutoff using UPARSE (version7.1 http://drive5.com/uparse/), and chimeric sequences were identified and removed using UCHIME. The taxonomy of each 16S rRNA gene sequence was analyzed by the RDP Classifier algorithm (http://rdp.cme.msu.edu/) against the Silva 16S rRNA database (silva 132/16s bacteria) using a confidence threshold of 70%.

### Metabolites sample preparation

Samples stored at −80 °C were taken out and thawed at room temperature. The following steps were conducted by Majorbio Bio-Pharm Technology Co., Ltd (Shanghai, China). In the experiment, 50 mg samples were used, and 400 μL methanol-water (4:1, v/v) were also added to homogenize the sample using a homogenizer for 10 s. The solution was ultrasonically extracted on ice for 10 min and stored at −20 °C for 30 min, then centrifuged for 15 min at 13000 rpm at 4 °C. For LC-MS analysis, 200 μL supernatant was used. QC sample was prepared by mixing aliquots of all samples to be a pooled sample and then analyzed using the same method with the analytic samples. The QCs were injected at regular intervals (every 10 samples) throughout the analytical run to provide a set of data from which repeatability can be assessed.

### LC/MS analysis parameters

The following steps were conducted by Majorbio Bio-Pharm Technology Co., Ltd. (Shanghai, China). LC-MS was performed on AB Sciex TripleTOF 5600TM mass spectrometer system (AB SCIEX, USA). LC Conditions: Column: Acquity BEH C18 column (100 mm × 2.1 mm i.d., 1.7 µm; Waters, Milford, USA). Solvent: The column was maintained at 40 °C and separation was achieved using the following gradient: 5% B–30% B over 0–3 min, 30% B–95% B over 3–9 min, 95% B–95% B over 9–13.0 min; 95% B–5% B over 13.0–13.1 min, and 13.1–16 min holding at 5 % B at a flow rate of 0.40 mL/min, where B is acetonitrile: isopropanol 1:1 (0.1% (v/v) formic acid) and A is aqueous formic acid (0.1% (v/v) formic acid). Injection Volume was 20 μL. The mass spectrometric data was collected using an AB Sciex TripleTOF 5600TM mass spectrometer system equipped with an electrospray ionization (ESI) source operating in either positive or negative ion mode with a capillary voltage 1.0 kV, sample cone, 40 V, collision energy 6 eV. The source temperature was set at 120 °C, with a desolvation gas flow of 45 L/h. Centroid data was collected from 50 to 1000 m/z with a 30000 resolution.

### In vitro differentiation of naïve CD4^+^T to Th1 and Th2 induced by the supernatant of mice feces

We used a previously well-established protocol for the in vitro culture of fecal samples and collected the supernatant.^44–46^ Briefly, gut feces of 7-month-old mice were collected, put into the culture medium (with or without GV-971, final concentration of 100 µg/mL) and 96 deep-well plates (2 mL total volume) at 37 °C on the shaking bed (500 rpm) inside the anaerobic incubator for a total culture period of 24 h before we collected the gut-microbiota-derived metabolites-containing supernatant. Naïve CD4^+^ T cells were separated from the splenocytes of 8-month-old C57BL/6 female mice using the naïve CD4^+^ T cell Isolation Kit (Stem Cell, Cat No. 19765). A total of 0.5 × 10^6^ cells/well in 0.5 mL of RPMI-1640 medium were plated in 48-well anti-CD3 and anti-CD28 pre-coated plates, and the culture medium was supplemented with cytokines and blocking antibodies. Th0: 20 ng/mL mIL-2; Th1: 20 ng/mL mIL-2, 10 μL supernatant, 5000 ng/mL 11B11; Th2: 20 ng/mL mIL-2, 10 μL supernatant, 5000 ng/mL XMG1.2. After incubation at 37 °C in 5% CO_2_ for 5 days, cells were stimulated with Cell Activation Cocktail (Thermo fisher, #00-4975-03) in 5% CO_2_ at 37 °C for 4 h, washed twice with PBS, labeled with zombie yellow (Biolegend, # 423104) to exclude dead cells. Non-specific binding of immunoglobulin to the Fc receptors was blocked with anti-CD16/32 (Biolegend, #101320). Intracellular staining was performed with anti-CD4 (Biolegend, #100406), anti-IFNγ (Biolegend, #505826) and anti-IL4 (Biolegend, #504104) according to the manufactures’ protocal. Cells were acquired on a BD Aria III cytometer, and data were analyzed using FlowJo software.

### Immunohistochemistry

For 3,30-diaminobenzidine (DAB)-developed staining, sections were immunostained using a Leica BOND-RX Autostainer (Leica Microsystems) and Coverslipper CV5030 (Leica Microsystems) according to the manufacturer’s IHC protocol. Briefly, sections were submitted to heat-induced epitope retrieval with Epitope Retrieval solution 2 (ER2, AR9640, Leica Biosystems) for 20 min. Endogenous peroxidase activity was blocked with 3%-4% (v/v) hydrogen peroxide (part of DS9800, Leica Biosystems) for 10 min. Then, sections were incubated with blocking buffer (10% NGS in PBS with 0.3% Triton x-100) for 60 min. Finally, staining was performed using the Bond Polymer Refine Detection System (DS9800, Leica Biosystems) according to the manufacturer’s protocol. The primary antibody incubation time was 30 min. Sections were stained for activated microglia using rabbit anti-IBA1 antibody (1:1,000, cat# 019-19741, Wako), amyloid depositions using mouse anti-Aβ42 antibody (1:1,000; cat# 803003, Biolegend) and Tau phosphorylation using mouse anti-PHF-Tau & tangles -Thr231 antibody (1:300, cat# MN1040, Thermo Fisher). Stained slices were automatically scanned by a high-throughput bright field scanner (NanoZoomer 2.0HT, Hamamatsu), and images were obtained by NDP.scan 3.2 software (Hamamatsu). For fluorescent staining, slides were blocked by blocking buffer (10% NGS in PBS with 0.3% Triton x-100) at RT for 1 h, and then incubated in the primary antibody solution (Iba-1 1:1,000, Aβ42 1:1,000, Tau 1:300) overnight at 4 °C. After washing, slides were incubated with fluorescent anti-rabbit or anti-mouse secondary antibody (1:1000, Invitrogen) for 60 min at RT and further washed 3 times in PBS. Finally, slides were counterstained with DAPI (1:10000 in PBS, Sigma) for 5 min at RT, washed, sealed and stored at 4 °C for image acquisition. Representative fluorescence images were acquired by upright fluorescence microscope Zmager-m2 (Zeiss, Germany) under 10× objective using Zen software (Zeiss).

### Amino acid detection

A set of amino acid standard mixture solutions was prepared at a concentration range of 100–2000 μmol/L. A portion of 10 μL of each standard mixture solution or plasma sample was pipetted into the bottom of a tube, and then 70 μL of sodium borate buffer (200 mmol/L at pH 8.8) was added. After 20 μL of 6-aminoquinolyl-N-hydroxysuccinimidyl carbamate (AQC) (4 mg/mL in acetonitrile) was added, the tube was closed and heated for 10 min at 55 °C to form AQC–amino acid. The solution was then cooled down to room temperature and 2 μL portion of each solution was injected into the UPLC-ESI-MS system for amino acid analysis without further purification.

### Human subjects

The blood from MCI due to AD patients and healthy control was collected from a pilot study. MCI due to AD is defined in the NIA-AA 2011 clinical and research diagnostic criteria for MCI due to AD. The patients with MCI due to AD in this study must meet the following criteria. First, concern regarding a change in cognition. Second, impairment in one or more cognitive domains. Third, preservation of independence in functional abilities. Forth, not demented (NIA-AA 2011 clinical diagnosis criteria for MCI due to AD). All participants underwent a screening process that included a review of their medical history, physical and neurological examinations, laboratory tests, and MRI scans. The clinical assessment of mild cognitive impairment or dementia included neuropsychological tests, as well as behavioral and psychiatric interviews conducted by attending psychiatrists. AD patients recorded scores of < 4 on the Hachinski Ischemia Scale and showed no history of significant systemic or psychiatric conditions, or traumatic brain injuries that could compromise brain function. The Clinical Dementia Rating (CDR) and Montreal Cognitive Assessment (MoCA) were assessed for all of the participants. Based on the assessment, we retained MCI due to AD subjects and others were excluded such as those who had impairment in a single non-memory domain (single, non-memory domain MCI subtype) and those who had impairment in two or more domains (multiple domains, slightly impaired MCI subtype). Normal control subjects had no history of cognitive decline, neurological disorders, or uncontrolled systemic medical disorders. All subjects included in this study had no more than two lacuna ischemia (of diameter < 1 cm) as revealed by MRI fluid-attenuated inversion recovery (FLAIR) sequence.

The sample size for the first cohort (Fig. 5h, i) is 9 MCI due to AD patients and 18 healthy subjects. The sample size for the second cohort (Fig. 5j) is 22 MCI due to AD patients and 22 healthy subjects. A diagnosis of MCI was based on the following criteria, which were adapted from the MCI diagnostic criteria of Petersen: (1) memory complaints, preferably corroborated by a spouse or relative; (2) objective memory impairment; (3) normal general cognitive function; (4) intact activities of daily living; and (5) absence of dementia. The Ethics Committee of the Shanghai Mental Health Centre Institutional Review Board approved the study (Number: 2016-22R1). Informed consents were obtained from the subjects and the guardian of the subjects. Information about gender and age etc. are provided below.

First cohort

**Table:** 

Type	Sample ID	Age	Gender
Healthy	HC-01	75	F
Healthy	HC-02	71	F
Healthy	HC-04	69	F
Healthy	HC-05	73	F
Healthy	HC-08	63	M
Healthy	HC-09	62	M
Healthy	HC-10	68	M
Healthy	HC-11	61	M
Healthy	HC-12	68	M
Healthy	HC-13	75	F
Healthy	HC-14	70	F
Healthy	HC-15	68	M
Healthy	HC-16	62	M
Healthy	HC-17	75	M
Healthy	HC-18	65	F
Healthy	HC-19	64	F
Healthy	HC-20	64	F
Healthy	HC-22	63	F
MCI due to AD	MCI-02	61	F
MCI due to AD	MCI-03	79	M
MCI due to AD	MCI-04	71	F
MCI due to AD	MCI-05	72	F
MCI due to AD	MCI-06	80	F
MCI due to AD	MCI-07	65	M
MCI due to AD	MCI-09	61	M
MCI due to AD	MCI-10	69	M
MCI due to AD	MCI-14	77	M

Second cohort

**Table:** 

Category	Sample ID	Age	Gender
Healthy	HC-0149	73	M
Healthy	HC-0150	70	F
Healthy	HC-0153	63	F
Healthy	HC-0156	67	M
Healthy	HC-0158	71	F
Healthy	HC-0165	61	F
Healthy	HC-0168	62	F
Healthy	HC-0172	70	F
Healthy	HC-0176	71	F
Healthy	HC-0187	73	F
Healthy	HC-0191	65	M
Healthy	HC-0013	70	F
Healthy	HC-0043	81	M
Healthy	HC-0050	64	F
Healthy	HC-0051	66	M
Healthy	HC-0056	75	M
Healthy	HC-0068	67	F
Healthy	HC-0074	57	F
Healthy	HC-0076	60	F
Healthy	HC-0081	71	F
Healthy	HC-0089	57	F
Healthy	HC-0090	76	F
MCI due to AD	MCI-0169	65	F
MCI due to AD	MCI-0175	61	F
MCI due to AD	MCI-0211	69	M
MCI due to AD	MCI-0229	65	M
MCI due to AD	MCI-0240	59	F
MCI due to AD	MCI-0244	64	F
MCI due to AD	MCI-0257	61	F
MCI due to AD	MCI-0260	60	F
MCI due to AD	MCI-0282	66	F
MCI due to AD	MCI-0284	64	F
MCI due to AD	MCI-0287	75	F
MCI due to AD	MCI-0303	62	F
MCI due to AD	MCI-0319	63	F
MCI due to AD	MCI-0332	67	F
MCI due to AD	MCI-0374	61	M
MCI due to AD	MCI-0385	68	F
MCI due to AD	MCI-0401	57	F
MCI due to AD	MCI-0414	64	F
MCI due to AD	MCI-0001	75	M
MCI due to AD	MCI-0043	55	F
MCI due to AD	MCI-0061	63	F
MCI due to AD	MCI-0091	78	F

### Th1 cell staining in human samples

Two milliliters of peripheral blood from healthy people or patients were treated with red blood cells lysis buffer. A total of 1 × 10^6^ cells were collected and incubated in 100 μL of PBS containing Zombie Yellow Dye (Biolegend, #423104) for 15 min to label live cells. After washing, samples were stained with APC/Cy7-conjugated anti-human CD45 (Biolegend, #304014), Alexa Fluor 700-conjugated anti-human CD3 (Biolegend, #344822), FITC-conjugated anti-human CD4 (Biolgend, #300506), Brilliant Violet 421-conjugated anti-human CXCR3 (Biolgend, #353716) and APC-conjugated anti-human CCR6 (Biolgend, #353416) for 30 min. Stained cells were washed twice with PBS. Cells were acquired using FACS Fortessa X-20 flow cytometer (BD Biosciences, San Jose, CA). Live CD45^+^CD3^+^CD4^+^CXCR3^+^CCR6^-^ cells were gated as Th1 subsets as previously described.^59^ Data analysis were performed with flowjo software (version 10.5, LLC)

### In vitro differentiation induced by phenylalanine and isoleucine

As previously described,^60^ naïve CD4^+^ T cells were enriched from the splenocytes of 8-month-old C57BL/6 female mice using the Naïve CD4^+^ T cell Isolation Kit (Stem Cell, #19765). For cell differentiation assay, a total of 1 × 10^5^ cells/well in 0.2 mL of RPMI-1640 medium were plated in anti-CD3/CD28-coated 96-well plates (anti-CD3, 2 μg/mL; anti-CD28, 1 μg/mL), and the culture medium was supplemented with cytokines or blocking antibodies. Th0: mIL-2 (10 ng/mL); Th1: mIL-2 (10 ng/mL) and 11B11 (5000 ng/mL). Phenylalanine (final concentration, 1 mmol/L), isoleucine (final concentration, 1 mmol/L) and GV-971 (final concentration, 100 µg/mL) were added into the indicated wells, respectively. After incubation at 37 °C in 5% CO_2_ for 5 days, cells were stimulated with Cell Activation Cocktail (Thermo fisher, #00-4975-03) in 5% CO_2_ at 37 °C for 4 h, washed twice with PBS, and labeled with zombie yellow (Biolegend, #423104) to exclude dead cells. Non-specific binding of immunoglobulin to the Fc receptors was blocked with anti-CD16/32 (Biolegend, #101320). Intracellular staining was performed with anti-CD4 (Biolegend, #100406) and anti-IFNγ (Biolegend, #505830) according to the manufactures’ protocal. Cells were acquired on a BD Fortessa cytometer, and data were analyzed using FlowJo software.

### In vitro proliferation induced by phenylalanine and isoleucine

A total of 1 × 10^6^ naïve CD4^+^ T cells were dissolved with 3 mL of Th1 differentiation Medium (10 ng/mL of mIL-2, 20 ng/mL of mIL-12 and 5000 ng/mL of 11B11) and plated in anti-CD3/CD28-coated 60-mm culture dish (anti-CD3, 2 μg/mL; anti-CD28, 1 μg/mL). The cells were incubated in 5% CO_2_ at 37 °C for 5 days.

Next, flow cytometry was applied to gate live cells (Zombie yellow dye, Biolegend, #423104) and CD4^+^CXCR3^+^ Th1 cells were sorted by anti-CD4 (Biolegend, #100406) and anti-CXCR3 (Biolegend, #126522) staining. The viability of sorted Th1 cells was further confirmed by Trypan blue staining.

The sorted Th1 cells was stained with CellTrace (Thermo Fisher, #C34557) and plated in anti-CD3/CD28-coated microplate and cultured in RPMI-1640 medium in the presence of phenylalanine (final concentration, 1 mmol/L), isoleucine (final concentration, 1 mmol/L) and GV-971 (final concentration, 100 µg/mL). After incubation in 5% CO_2_ at 37 °C for 4 days, cells were stimulated with Cell Activation Cocktail (Thermo fisher, #00-4975-03) in 5% CO_2_ at 37 °C for 4 h, washed twice with PBS, and labeled with zombie yellow (Biolegend, #423104) to exclude dead cells. Non-specific binding of immunoglobulin to the Fc receptors was blocked with anti-CD16/32 (Biolegend, #101320). Intracellular staining was performed with anti-CD4 (Biolegend, #100422) and anti-IFNγ (Biolegend, #505806) according to the manufactures’ protocal. Cells were acquired on a BD Fortessa cytometer, and data were analyzed using FlowJo software.

### Uptake of phenylalanine

Naïve CD4^+^ T cells were separated from the splenocytes of 8-month-old C57BL/6 female mice using the Naïve CD4^+^ T cell Isolation Kit (Stem Cell, Cat No. 19765) and were induced to Th1 differentiation by 20 ng/mL IL-12. After 3 days, a total of 5 × 10^5^ cells/well Th1 cells in 0.5 mL of RPMI-1640 medium were plated into 48-well plates. ^13^C-labelled phenylalanine and 5 mM amino transporter inhibitor BCH (CAS:20448-79-7) were added into indicated wells. After 0.5 h, cells were collected and washed twice with ice-cold D-PBS. Then, 50 µL deionized water was added and cells were lysed through freezing and thawing twice at −80 °C. The cell lysate was centrifuged at 12000 × *g* for 10 min and ^13^C-labelled phenylalanine in the supernatant was analyzed by Mass spectrometry.

### Antibiotic treatments

Mice were treated by adding an antibiotic solution (ABX) containing ampicillin (0.1 mg/mL, final concentration in drinking water), streptomycin (0.5 mg/mL, final concentration in drinking water), and colistin (0.1 mg/mL, final concentration in drinking water) (Sigma-Aldrich) to sterile drinking water. Solutions and bottles were changed 3 times and once weekly, respectively. The antibiotic activity was confirmed by 16S rRNA gene sequencing. Types of bacteria with a relative abundance of less than 0.01 are deleted in the Tg group. The duration of ABX treatment was slightly different based on the experimental settings. In the context of faecal microbia transplantation experiments, mice received 3 days of ABX before undergoing faecal microbia transplantation the next day by oral gavage using animal feeding needles.

### FMT experiments

FMT was performed by thawing faecal material. Then, 200 μL of the suspension was transferred by oral gavage into each ABX-pretreated recipient. Twelve-month-old C57 mice were first treated with an antibiotic cocktail in drinking water for 3 days, and then 40 mg of the mixed stool suspended in PBS was inserted by gavage into each mouse 3 times with a 2-day break in between. After 3 days, 4.2 µg Aβ 1–40 oligomer was injected into both sides of the hippocampus. The mice were sacrificed 3 days later and used for different analyses.

### Bioinformatics analysis

Principal component analysis (PCA) is a method that simplifies the complexity in high-dimensional data while retaining trends and patterns. PCA reduces data by geometrically projecting them onto lower dimensions called principal components (PCs), with the goal of finding the best summary of the data using a limited number of PCs.^28^ Principle coordinate analysis (PCoA) is the modified version of PCA analysis, in which different distance metrics (e.g., Bray-Curtis distances) can be used, compared to the Euclidean distances that is solely used in PCA. Both PCA and PCoA analysis used in this study were performed at Majorbio I-Sanger Cloud platform (www.i-sanger.com). For correlational analysis, trend changes in the abundance of bacteria were correlated with changes in the frequency of immune cells. The exact correlation coefficient and the p-value (set to 0.05) were calculated using the Pearson parametric correlation test using the R package “ggcorrplot”. Correlational circus maps were generated using the R package “igraph”. Pathway analysis and biological function enrichment analysis were performed using the Kyoto Encyclopedia of Gene Genotype (KEGG). Data were enriched using the R package “DOSE”, “GO.db”, “GSEABase” and “ClusterProfiler”. Only pathways with a false discovery rate (FDR) corrected p-value of < 0.05 were represented. Bacterial stream plots were performed using the R package “ggalluvial”. The k-means algorithm was used for clustering different types of immune cells to the cluster with the nearest mean along the time course using the R package “TCseq” *(Wu M, Gu L (2019). TCseq: Time course sequencing data analysis. R package version 1.8.0)*. A standardized z-score transformation was applied to convert the fraction values to z-scores before analysis in all heatmaps. Heatmaps and volcano plots are also plotted using the R packages. The ROC biomarker analysis and random forest classification were performed with MetaboAnalyst 4.0 (https://www.metaboanalyst.ca/). The Mouseac database used in Supplementary information, Fig. S2 is http://www.mouseac.org/. Other bioinformatics analyses, including the barplot representation of gut microbiota abundance at the family level, lists of significant changing bacteria, etc. were conducted using the online platform of the Majorbio I-Sanger Cloud (www.i-sanger.com).

### Flow cytometry

Mice were anesthetized, blood samples were collected into EDTA-containing tubes, and red blood cells were removed using 1 × red blood lysis buffer. Before tissue collection, the brains were perfused with ice-cold PBS to avoid sampling the circulating blood immune cells, and the brains were removed, chopped into pieces and dissected according to the introduction of the Adult Brain Dissociation Kit (Miltenyi, Cat No. 130-107-677) using the gentleMACS dissociator (Miltenyi Biotec). The brain homogenate was filtered through a 70-μm cell strainer and centrifuged at 300 × *g* for 5 min at 4 °C. The cell pellet was resuspended in cold PBS buffer and centrifuged again at 300 × *g* for 5 min at 4 °C. All samples were counted and adjusted to a density of 2–3 × 10^6^/100 μL, labeled with a Live/Dead kit for 30 min, and then centrifuged at 500 × *g* for 3 min at 4 °C. The cells were resuspended in 100 μL PBS buffer, blocked with anti-CD16/32 (Biolegend, Cat No. 101320) for 10 min, and incubated with the antibody according to the manufacturers’ protocols at 4 °C for 30 min. The following antibodies were used in the FACS analysis: CD45(30-F11)-APC-Cy7(103116, Biolegend), CD11b(M1/70)-FITC (101205, Biolegend), CX3CR1(SA011F11)-PE-Dazzle 594 (149014, Biolegend), Siglec-H-BV421 (566581, BD), F4/80(BM8)-BV421 (123132, Biolegend), CD86(IT2.2)-PE (305438, Biolegend), CD206(15-2)-APC (321110, Biolegend), CD206(15-2)-BV785 (321142, Biolegend), CD11c(N418)-PE-Cy7 (117318, Biolegend), CD8(53-6.7)-Percp-Cy5.5 (100734, Biolegend), Ly-6C(HK1.4)-PE-Dazzle 594 (128044, Biolegend), Gr-1(RB6-8C5)-Percp-Cy5.5 (108428, Biolegend), B220(RA3-6B2)-BV421 (103240, Biolegend), CD19(6D5)-PE (115508, Biolegend), CD49b(DX5)-PE-Cy7 (108922, Biolegend), CD4(GK1.5)-PE-Cy7 (100422, Biolegend), CD4(GK1.5)-FITC (100406, Biolegend), CXCR3(CXCR3-173)-BV421 (126522, Biolegend), CCR4(L291H4)-PE-Cy7 (359410, Biolegend), CCR6(29-2L17)-APC (129814, Biolegend), CD127(A019D5)-PE (351304, Biolegend), CD25(3C7)-Percp-Cy5.5 (101912, Biolegend), Live/Dead (423104, Biolegend). Cells were added to 500 μL PBS buffer, centrifuged at 500 × *g* for 3 min at 4 °C and resuspended in 200 μL running buffer. Relevant negative control, Fluorescence Minus One (FMO) control and each fluorescence compensation sample were used to adjust fluorescence compensation and identify the populations of interest. Cells were acquired on a BD Aria III cytometer, and data were analyzed using FlowJo software.

### Antibody array

The following steps were conducted by RayBiotech (Guangzhou, China). The brain homogenates (from 20 mg tissue) were analyzed with a glass slide-based antibody cytokine array including 200 proteins (RayBiotech, GSM-CAA-4000-1). A 100 μL sample diluent was added to each well and incubated at room temperature for 30 min. Then, the buffer was replaced with another 100 μL of sample and incubated at room temperature for 2 h. The samples were discarded and washed 5 times (5 min each) with 150 μL of 1 × Wash Buffer I and 2 times (5 min each) with 150 μL of 1 × Wash Buffer II at room temperature with gentle shaking. After that, 80 μL of the detection antibody cocktail were added to each well and incubated at room temperature for 2 h. The slide was washed 5 times (5 min each) with 150 μL of 1 × Wash Buffer I and then 2 times with 150 μL of 1 × Wash Buffer II at room temperature with gentle shaking. Eighty microliters of Cy3 equivalent dye-conjugated streptavidin was added to each well and incubated at room temperature for 1 h. After 5 washes (5 min each), the signal was visualized through a laser scanner. The data were then visualized by a heatmap diagram (www.metaboanalyst.ca).

### Brain section preparation

Mice were transcardially perfused with 0.9% NaCl after deep anesthesia with pentobarbital (100 mg/kg, i.p.). Brain tissues were quickly removed, frozen and stored at −70 °C. Serial coronal brain sections (12 μm thickness) were created using a sliding, freezing microtome (Leica Microsystems), mounted on slides and dried overnight in the air at room temperature. Tissue sections were stored at −80 °C or used immediately.

### Laser microdissection and Q-PCR analysis

Frozen mouse brain samples were sectioned and collected on PEN membrane slides (Leica, 11600288). The hippocampus was isolated by laser microdissection microscopy (Leica Microsystems, LMD6). RNA was extracted with a RNeasy Micro Kit (Qiagen, 74004) and reverse transcribed into cDNA (Takara, PrimeScript RT Master Mix, RR036A). Q-PCR was performed using the ABI 7500 system via the SYBR method (Takara, SYBR Premix Ex Taq II). Following primers were used: Synaptophysin-forward: CAGTTCCGGGTGGTCAAGG; Synaptophysin-reverse: ACTCTCCG TCTTGTTGGCAC; actin-forward: GCTCTTTTCCAGCCTTCCTT; actin-reverse: AGGTCTTTACGGAT GTCAACG.

### Statistical analysis

In the behavior test, animals were randomly distributed into different groups. For two group comparisons, an unpaired two-tailed Student’s t-test was applied. For significantly changing bacteria lists, we used the online platform of the Majorbio I-Sanger Cloud to perform Wilcoxon rank-sum test, and the *P*-value was based on a two-tailed test with FDR corrected, the significant level was set to 0.05, and the 0.95 confidence intervals were calculated through the bootstrap algorithm. For more than two group comparisons, one-way ANOVA or two-way ANOVA followed by Dunnett’s test was performed. All data with error bar are represented as mean ± SEM. *P* < 0.05 was considered statistically significant. Most of the data were analyzed in GraphPad Prism. For image quantification, IBA-1-positive, Aβ 42-positive and phosphorylated Tau-positive cells were analyzed by ImageJ v1.8.0 with ‘area’ readout.

## Supplementary information

Supplementary figure S1

Supplementary figure S2

Supplementary figure S3

Supplementary figure S4

Supplementary figure S5

supplementary table S1

supplementary table S2

supplementary table S3

supplementary table S4

supplementary table S5

supplementary table S6
