# Supplementary material for: Sodium oligomannate therapeutically remodels gut microbiota and suppresses gut bacterial amino acids-shaped neuroinflammation to inhibit Alzheimer’s disease progression
Source: Cell Res. 2019 Sep 6;29(10):787–803. doi: 10.1038/s41422-019-0216-x (PMC6796854; doi:10.1038/s41422-019-0216-x)
Supplement: Supplementary file 1 — Supplementary figure S1 [file 41422_2019_216_MOESM1_ESM.pdf]

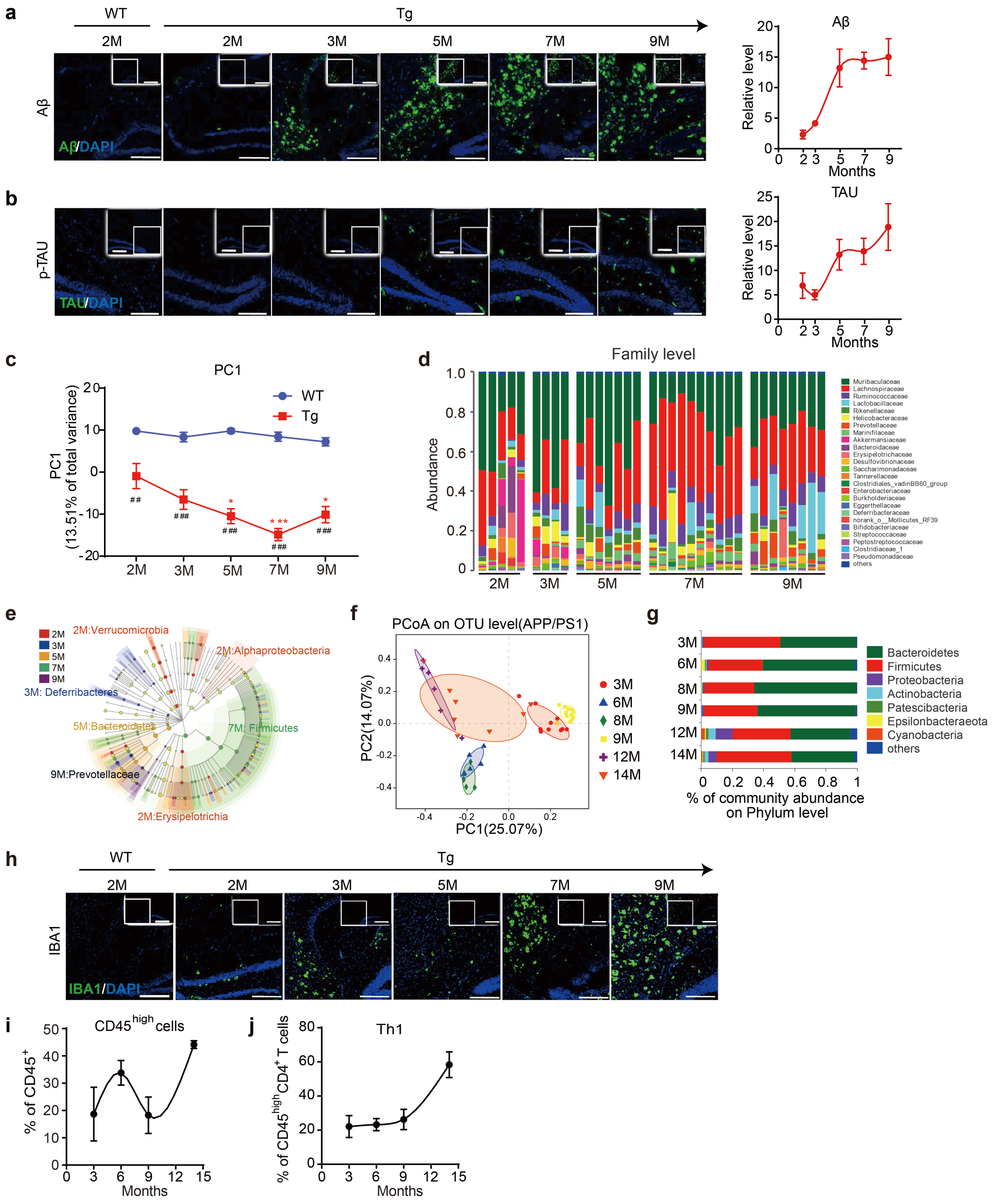

Figure S1

(a-b) Changes in Aβ positive area (a) and phosphorylated-tau (p-TAU) positive area (b) in hippocampal slices from 5XFAD transgenic (Tg) mice (2-, 3-, 5-, 7-, and 9-month-old) versus age-paired wild-type (WT) mice (n = 2-7). Representative fluorescent images are presented; the scale

bar represents 500 μm in the small bracket (upper), 250 μm in the large bracket (down). Line charts summarize the results from all individual points relative to WT mice and are presented as the mean ± standard error of the mean (mean ± sem). Lines are fitted with a cubic spline algorithm. M, months.

(c) Principal component (PC) 1 from principal component analysis (PCA) of the gut microbiome composition at the operational taxonomic unit (OTU) level in WT and 5XFAD transgenic (Tg) mice (2-, 3-, 5-, 7-, and 9-month-old) (n=4-10). Red points and lines, Tg mice; blue points and lines, WT mice. Lines are connected naturally without any fitting. The unpaired parametric t-test is used; *indicates a significant difference compared to 2-month-old in Tg. *$P = 0.0146$, ***$P = 0.0004$, *$P = 0.02$. #indicates significant difference compared to age-matched WT mice. ##$P = 0.032$; ###$P < 0.0001$. M, months.

(d) Changes in the relative abundance of gut microbes at the family level in 5XFAD transgenic (Tg) mice (2-, 3-, 5-, 7-, and 9-month-old) (n=4-10). Different colours represent different families.

(e) Cladogram of linear discriminant analysis effect size (LEfSe) analysis of the gut microbiome composition of 5XFAD transgenic (Tg) mice (2-, 3-, 5-, 7-, and 9-month-old) (n=4-10). The bacteria with the highest discriminatory power are labelled on the graph at each month. Colours indicate bacteria taxa that are enriched in each month. The inner to outer circles indicate different taxonomic levels (inner to outer: phylum, class, order, family and genus). M, months.

(f) PCA analysis of the gut microbiome at the OTU level in APP/PS1 transgenic mice at 3-, 6-, 8-, 9-, 12- and 14-month-old (n=4-12). Colours and shapes indicate data from different months. Coloured ellipses indicate 0.95 confidence interval (CI) ranges within each tested group. PC, principal component.

(g) Changes in the relative abundance of gut microbes at the phylum levels in APP/PS1 transgenic mice at 3-, 6-, 8-, 9-, 12- and 14-month-old (n=4-12). Colours represent different phyla. M, months.

(h) Representative fluorescent images of changes in IBA1 positive are in hippocampal slices from 5XFAD transgenic (Tg) mice (2-, 3-, 5-, 7-, and 9-month-old) versus 2-month-old wild-type (WT) mice (n=2-7). The scale bar represents 500 μm in the small bracket (upper), 250 μm in the large bracket (down). M, months.

(i) Changes in the frequency of infiltrating cells ($CD45^{high}$) detected in the whole-brain homogenates of APP/PS1 mice at 3-, 6-, 9- and 14-month-old as detected by flow cytometry (n=5-8). Cell counts are presented relative to the frequency of $CD45^{+}$ cells and formatted as the mean ± standard error of the mean (mean ± sem). Lines are fitted with a cubic spline algorithm.

(j) Changes in the frequency of infiltrating peripheral Th1 cells in the whole-brain homogenates of APP/PS1 mice at 3-, 6-, 9- and 14-month-old as detected by flow cytometry (n = 3-8). Th1 cells ($CD45^{high}CD4^{+}CXCR3^{+}$) are presented relative to the frequency of $CD45^{high}CD4^{+}$T cells. The data are presented as the mean ± standard error of the mean (mean ± sem). Lines are fitted with a cubic spline algorithm.
