# Supplementary material for: Sodium oligomannate therapeutically remodels gut microbiota and suppresses gut bacterial amino acids-shaped neuroinflammation to inhibit Alzheimer’s disease progression
Source: Cell Res. 2019 Sep 6;29(10):787–803. doi: 10.1038/s41422-019-0216-x (PMC6796854; doi:10.1038/s41422-019-0216-x)
Supplement: Supplementary file 2 — Supplementary figure S2 [file 41422_2019_216_MOESM2_ESM.pdf]

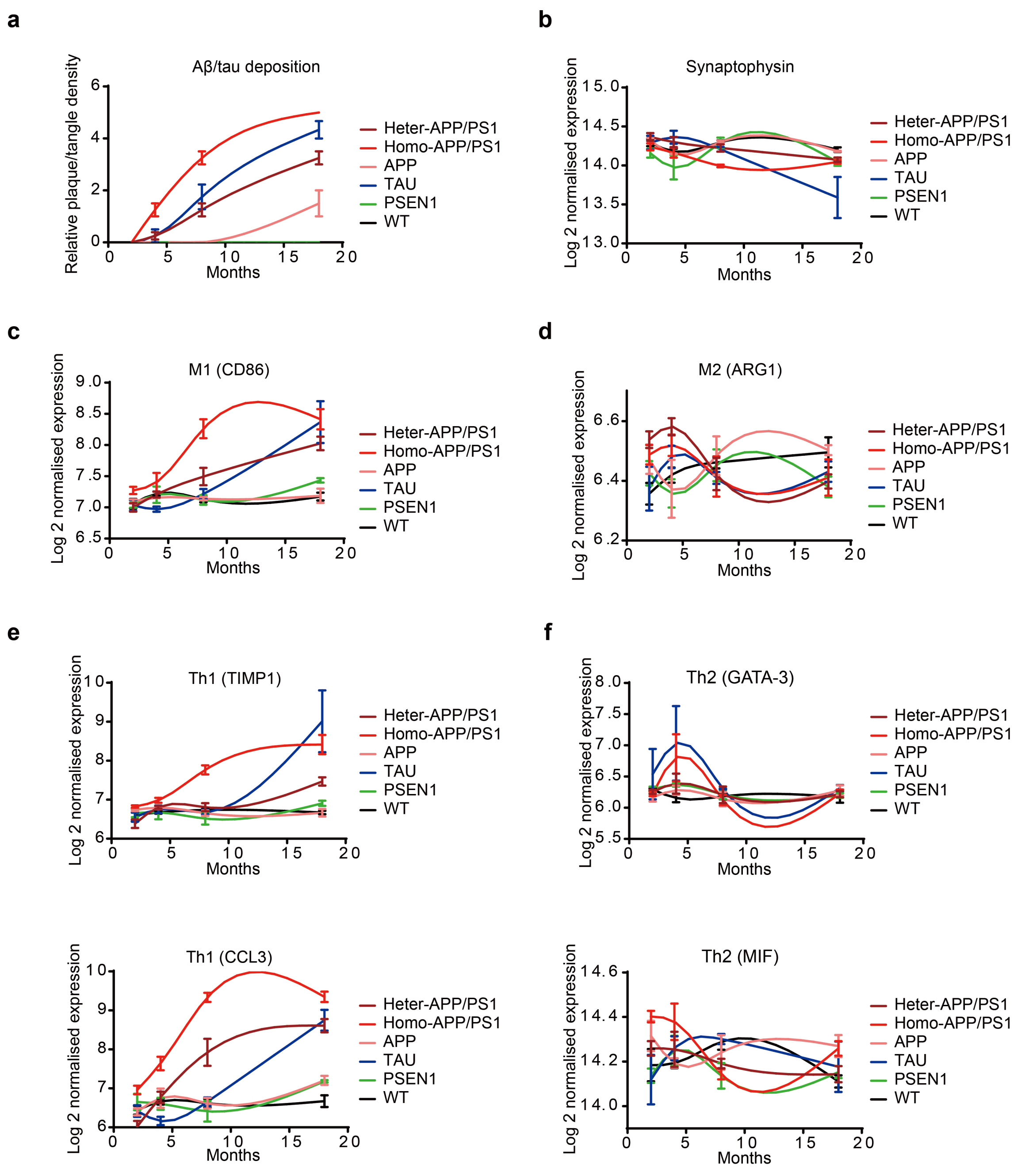

Figure S2. Changes in the hallmarks of AD in five animal models and wild-type (WT) mice at 2-, 4-, 8- and 18-month-old from the Mouseac database.

(**a**) Changes in relative densities of Aβ plaque or tau neurofibrillary tangle (n = 4 for each group). The data are presented as the mean ± standard error of the mean (mean ± sem). Colours indicate different models; lines are fitted using a polynomial algorithm.

(**b**) Changes in log_2 normalized expression levels of synaptophysin (n = 4 for each group). The data are presented as the mean ± standard error of the mean (mean ± sem). Colours indicate different models; lines are fitted using a polynomial algorithm.

(**c-d**) Changes in log_2 normalized expression levels of CD86 and ARG1, representing changes in M1 and M2 cell counts (n = 4 for each group). The data are presented as the mean ± standard error of the mean (mean ± sem). Colours indicate different models; lines are fitted using a polynomial algorithm.

(**e-f**) Changes in log_2 normalized expression levels of TIPM, CCL3, GATA-3 and MIF, representing changes in Th1 and Th2 counts (n = 4 for each group). The data are presented as the mean ± standard error of the mean (mean ± sem). Colours indicate different models; lines are fitted using a polynomial algorithm.
