# Supplementary material for: Sodium oligomannate therapeutically remodels gut microbiota and suppresses gut bacterial amino acids-shaped neuroinflammation to inhibit Alzheimer’s disease progression
Source: Cell Res. 2019 Sep 6;29(10):787–803. doi: 10.1038/s41422-019-0216-x (PMC6796854; doi:10.1038/s41422-019-0216-x)
Supplement: Supplementary file 3 — Supplementary figure S3 [file 41422_2019_216_MOESM3_ESM.pdf]

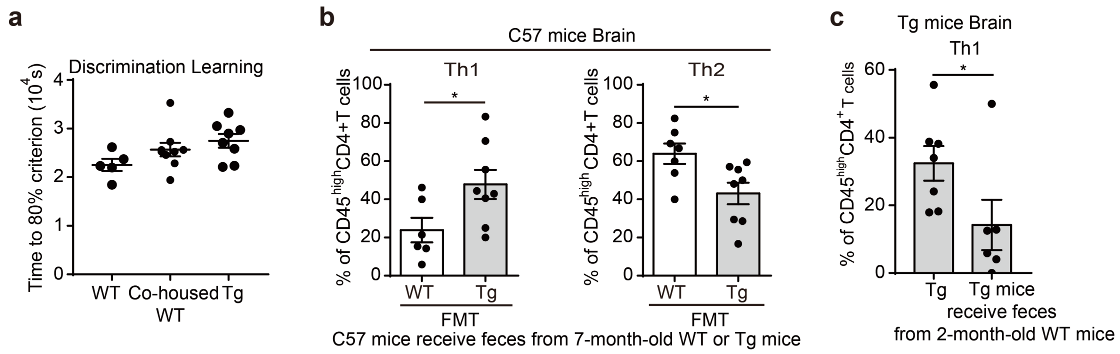

Figure S3.

(a) Effects of co-housing on the time out of 10^4 seconds taken to achieve 80% success in a test to evaluate the discrimination learning abilities of WT, co-housed WT and 5XFAD transgenic (Tg) mice at 7-month-old (n = 5-8). Time spent means the time to reach the 80% performance level (seconds); the higher the time spent, the more severe the cognitive impairment is (see Methods).

(b) Effects of faecal microbiota transplantation (FMT) on the frequency of brain immune cells (Th1 and Th2) in 12-month-old C57 recipient mice injected with Aβ (see Methods) using feces of either 9-month-old WT or 5XFAD transgenic (Tg) mice. Th1 cells ($CD45^{high}CD4^{+}CXCR3^{+}$) and Th2 cells ($CD45^{high}CD4^{+}CCR4^{+}$) are presented relative to $CD45^{high}$ $CD4^{+}$T cells (n = 6-8); the data are presented as the mean ± standard error of the mean (mean ± sem). *P = 0.041 (Th1), *P = 0.020 (Th2) by Student's t-test. C57, the C57BL/6 wild type (WT) mice.

(c) Effects of faecal microbiota transplantation (FMT) on the frequency of brain Th1 cells in 7-month-old 5XFAD (Tg) recipient mice injected with Aβ (see Methods) using feces of 2-month-old WT mice (n = 6-7). The data are presented as the mean ± standard error of the mean (mean ± sem). *P = 0.035 by Student's t-test.
