# Supplementary material for: Sodium oligomannate therapeutically remodels gut microbiota and suppresses gut bacterial amino acids-shaped neuroinflammation to inhibit Alzheimer’s disease progression
Source: Cell Res. 2019 Sep 6;29(10):787–803. doi: 10.1038/s41422-019-0216-x (PMC6796854; doi:10.1038/s41422-019-0216-x)
Supplement: Supplementary file 4 — Supplementary figure S4 [file 41422_2019_216_MOESM4_ESM.pdf]

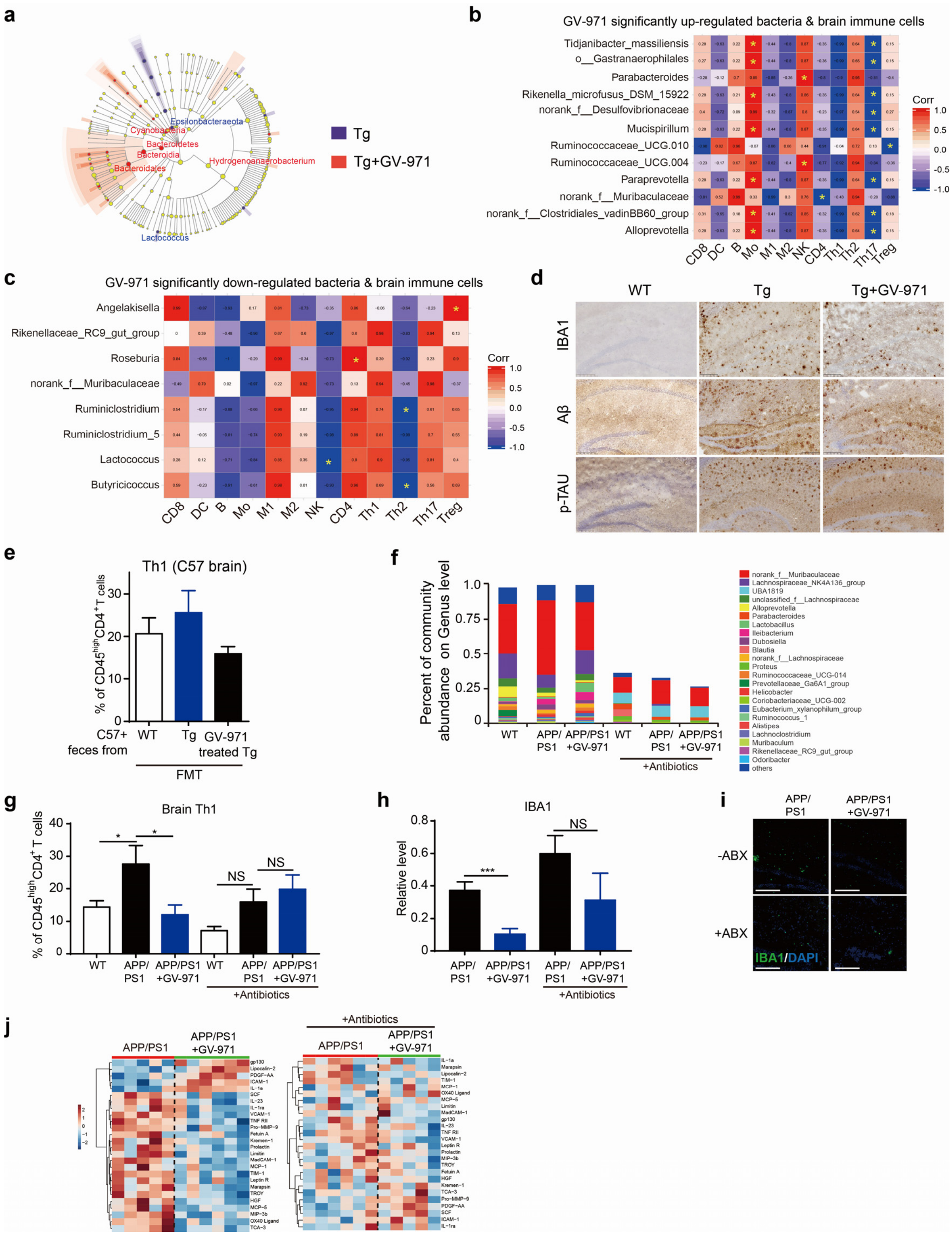

Figure S4.

(a) Cladogram of linear discriminant analysis effect size (LEfSe) analysis of the gut microbiome

composition of 7-month-old 5XFAD transgenic (Tg) mice treated orally with 100 mpk GV-971 (n=5-7). The phylum, class, order or genus level of represented bacteria with the highest discriminatory power of each group are labelled on the graph. Blue, bacteria enriched in 7-month-old Tg mice. Red, bacteria enriched in 7-month-old Tg mice that received 100 mpk GV-971. The inner to outer circles indicate different taxonomic levels (inner to outer: phylum, class, order, family and genus).

(b-c) Correlation of trend changes between the abundance of microbiota that are significantly up- (b) and down-regulated (c) by 100 mpk GV-971 and the frequency of brain immune cell subtypes in 5XFAD transgenic (Tg) mice at 7-month-old, represented at genus level. Related to Figure 4b. Squares in red (positive correlation) or blue (negative correlation) with a yellow asterisk (*) indicate significant correlations with $P < 0.05$ measured by the Pearson parametric correlation test, the numbers in each cell are correlation coefficient (see Methods). DC, dendritic cells; B, B cells; Mo: monocytes; NK, natural killer cells; Treg, T regulatory cells.

(d) Representative images of IBA1 staining, Aβ deposition and Tau phosphorylation in the brain hippocampus of WT, Tg and GV-971-treated Tg. Scale bar represent 250 μm. Positive signal was visualized using the substrate 3,3' diaminobenzidine (DAB) shown as dark brown.

(e) Effects of feces from WT, Tg and 100 mpk GV-971-treated Tg mice on the brain Th1 cell in the recipient C57 mice with Aβ hippocampus injection (n = 4-5). The donor of the feces are all 7-month-old. The data are presented as the mean ± standard error of the mean (mean ± sem). FMT, fecal microbiota transplantation. C57, the C57BL/6 mice.

(f) Effects of antibiotic treatment (ampicillin (0.1 mg/mL), streptomycin (0.5 mg/mL), and colistin (0.1 mg/mL) on the relative abundance of gut microbes on the genus level in 6-month-old APP/PS1 transgenic model mice treated orally with 50 mpk GV-971 (n = 6-8). Colours indicate different genera. f, family.

(g) Effects of 50 mpk GV-971 on the brain Th1 cell frequency of antibiotic-treated 6-month-old

APP/PS1 mice (see Methods). Th1 cells ($CD45^{high}CD4^{+}CXCR3^{+}$) are presented relative to $CD45^{high}$ $CD4^{+}$T cells (n = 6-8), and the data are presented as the mean ± standard error of the mean (mean ± sem). From left to right: *P = 0.0444, *P = 0.0291; P = 0.0650 (NS), P = 0.0931 (NS) by Student's t-test. NS, no significance.

(h) Effects of 50 mpk GV-971 on the relative density of IBA1-positive immune-fluorescent staining detected in hippocampal slices from antibiotic-treated 6-month-old APP/PS1 mice (n = 4-6, see Methods). The IBA1-positive area reflects the activation of microglial cells. The data are presented as the mean ± standard error of the mean (mean ± sem). ***P < 0.0001 by Student's t-test. NS, no significance.

(i) Representative immunofluorescence staining of IBA1 in the brain of APP/PS1 and GV-971 treated APP/PS1 mice with or without antibiotics. IBA1 was visualized using FITC conjugated secondary antibody shown as green. Nucleus were stained with DAPI shown as blue. Scale bar represent 250 μm.

(j) Effects of GV-971 on cytokine levels in the brain homogenates of 8-month-old APP/PS1 mice as detected by a cytokine antibody array with and without antibiotics treatment (n = 5-6). Colours in the heatmap indicate relative cytokine levels; red indicates cytokines that are upregulated, and blue indicates cytokines that are downregulated.
