# Supplementary material for: Sodium oligomannate therapeutically remodels gut microbiota and suppresses gut bacterial amino acids-shaped neuroinflammation to inhibit Alzheimer’s disease progression
Source: Cell Res. 2019 Sep 6;29(10):787–803. doi: 10.1038/s41422-019-0216-x (PMC6796854; doi:10.1038/s41422-019-0216-x)
Supplement: Supplementary file 5 — Supplementary figure S5 [file 41422_2019_216_MOESM5_ESM.pdf]

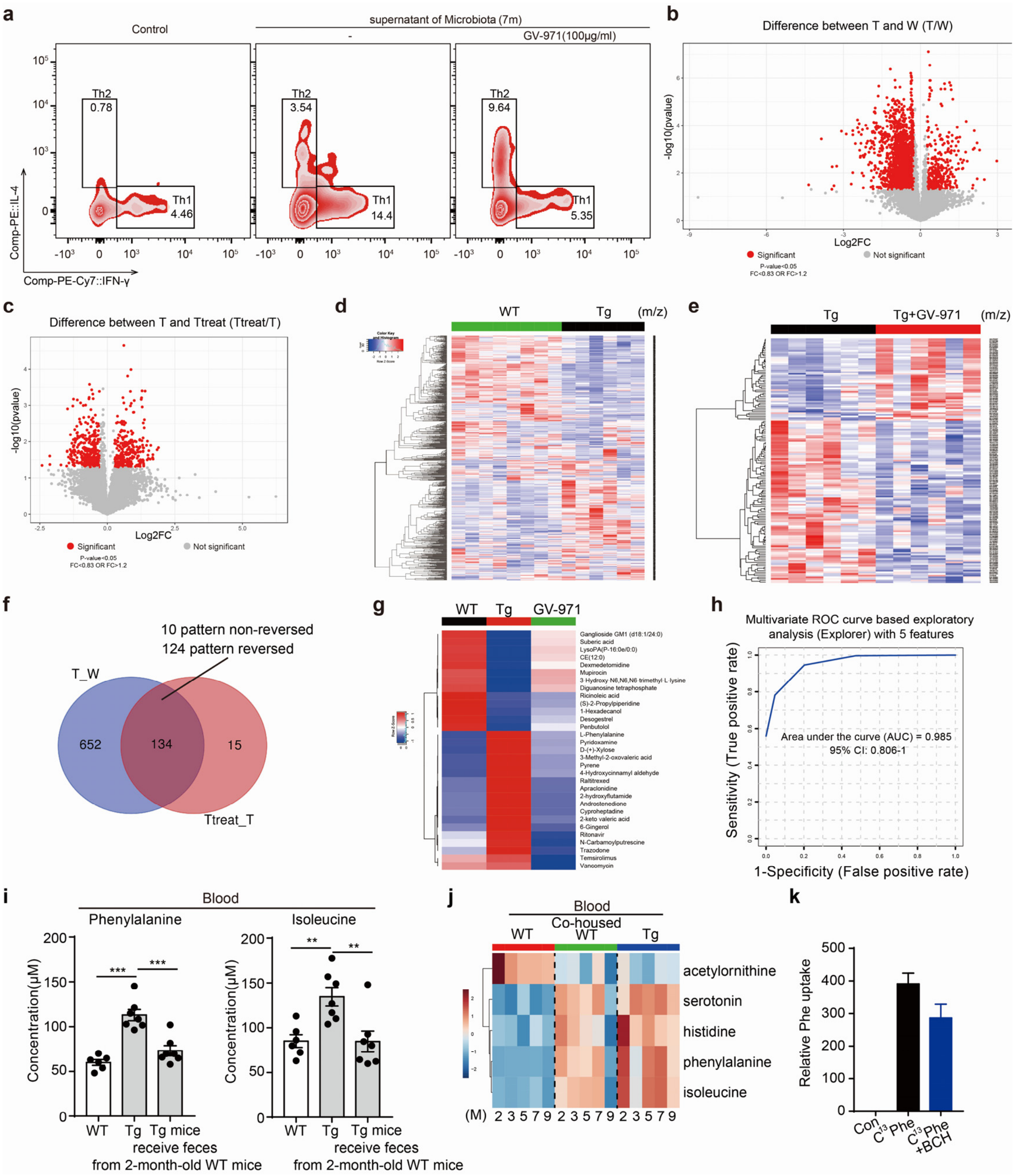

Figure S5.

(a) The effect of GV-971 treated bacteria supernatant on the differentiation of naïve CD4^{+}T cells to Th1 and Th2 cells *in vitro*. Th1 (CD4^{+}IFN-γ^{+}) cells and Th2 (CD4^{+}IL-4^{+}) cells were gated by flow cytometry. The data are presented as the mean ± standard error of the mean (mean ± sem); n = 3 replicates per group, one of three replicated results was represented.

(b-c) The volcano plot depicts the fold change and *P*-value distribution of metabolites from the raw data of gut feces metabolomics of 7-month-old WT and 5XFAD (Tg) mice (n=6-8) (b) and 7-month-old 5XFAD (Tg) mice treated or not treated with 100 mpk GV-971 (n=6) (c). Red points indicate significant changing metabolites. Significance is defined as *P*-value < 0.05 of Student's t-test and a fold change (FC) of < 0.83 or > 1.2 between Tg (T) and wild type (W), or between Tg (T) **(b)** and Tg treated with GV-971 (Ttreat) **(c)**. The x-axis shows $\log_2$FC, and the y-axis shows $-\log_{10}P$. T, 5XFAD (Tg) mice; W, wild type mice; Ttreat, 5XFAD mice treated with 100 mpk GV-971.

(d-e) Heatmap of seven hundred eighty-six metabolites that were differentially regulated between Tg and WT mice (d), as well as 149 metabolites between GV-971-treated and untreated Tg mice (e) (n=6-8). These metabolites were identified and annotated by aligning the molecular mass data (m/z) of the significant peaks with online METLIN database.

(f) The Venn diagram shows the commonly deregulated gut feces metabolites between Tg and WT mice (T_W) and between GV-971-treated and untreated Tg mice (Ttreat_T) (n=6-8). One hundred twenty-four metabolites had reversed patterns across the two comparisons, i.e., metabolites that are either both high in T versus W (T_W) and low in Ttreat versus T (Ttreat_T), or both low in T versus W (T_W) and high in Ttreat versus T (Ttreat_T). T, 5XFAD (Tg) mice; W, wild type mice; Ttreat, 5XFAD mice treated with 100 mpk GV-971.

(g) The heatmap shows 31 identified metabolites that were differentially regulated among the WT, Tg and 100 mpk GV-971-treated Tg groups (n=6-8) that could be matched to all three

databases (Human Metabolites Database (HMDB), METLIN, Kyoto Encyclopedia of Genes and Genomes (KEGG)). Red, upregulated; blue, downregulated.

(h) The receiver operating characteristic (ROC) curve analysis shows that the model containing the best 5 biomarkers had a strong diagnostic power (with an AUC of 0.985) in discriminating Tg from WT.

(i) Effects of faecal microbiome transplantation on blood phenylalanine and isoleucine levels. Feces of 2-month old WT mice were transplanted into 7-month old Tg mice (n = 6-7). For phenylalanine, ***P = 0.0001((F (2, 17) = 26.59)). For isoleucine, **P = 0.0065 (Tg versus WT), **P = 0.0045 (Tg receive feces versus Tg) by one-way ANOVA (F (2, 17) = 8.181).

(j) Levels of amino acid in the blood samples of WT, co-housed WT and Tg at various months (M) of ages. Red, upregulated; blue, downregulated.

(k) The uptake of phenylalanine by Th1 cells. Th1 cells were cultured with/without ^{13}C-labelled phenylalanine (5 mM) for 0.5 h. Cells were collected for mass spectrum analysis to test the relative concentration of ^{13}C-labelled phenylalanine. BCH, an inhibitor of system L amino acid transporters, was used as positive control. n = 3 per replicate.
